# Luminescent supramolecular hydrogels from a tripeptide and nitrogen-doped carbon nanodots

**DOI:** 10.3762/bjnano.8.157

**Published:** 2017-08-01

**Authors:** Maria C Cringoli, Slavko Kralj, Marina Kurbasic, Massimo Urban, Silvia Marchesan

**Affiliations:** 1 Department of Chemical and Pharmaceutical Sciences, University of Trieste, Via L. Giorgieri 1, Trieste 34127, Italy,; 2 Department for Materials Synthesis, Jožef Stefan Institute, Jamova 39, Ljubljana 1000, Slovenia

**Keywords:** carbon nanodots, composites, hydrogels, nanomaterials, peptide self-assembly

## Abstract

The combination of different components such as carbon nanostructures and organic gelators into composite nanostructured hydrogels is attracting wide interest for a variety of applications, including sensing and biomaterials. In particular, both supramolecular hydrogels that are formed from unprotected D,L-tripeptides bearing the Phe-Phe motif and nitrogen-doped carbon nanodots (NCNDs) are promising materials for biological use. In this work, they were combined to obtain luminescent, supramolecular hydrogels at physiological conditions. The self-assembly of a tripeptide upon application of a pH trigger was studied in the presence of NCNDs to evaluate effects at the supramolecular level. Luminescent hydrogels were obtained whereby NCND addition allowed the rheological properties to be fine-tuned and led to an overall more homogeneous system composed of thinner fibrils with narrower diameter distribution.

## Introduction

**Scheme 1:**
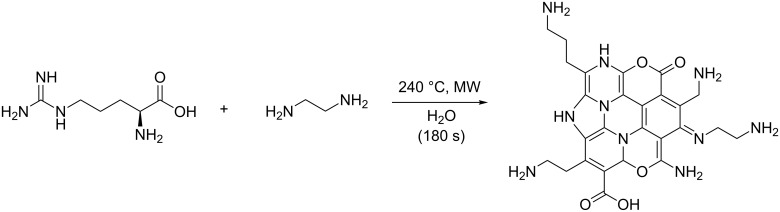
Microwave-assisted formation of nitrogen-doped carbon nanodots from arginine [6].

Carbon nanodots (CNDs) are quasi-spherical nanoparticles with a diameter less than 10 nm. They are a very interesting class of nanocarbons because of their excellent water solubility, ease of functionalization, high chemical stability and resistance to photo-bleaching. In particular, CNDs have attracted particular interest in light of their biocompatibility, combined with their fascinating fluorescence properties, such as excitation-dependent emission range. Their properties allow them to have an important impact in biological and environmental applications as alternatives to traditional, toxic, semiconductor-based quantum dots (QDs). They can be employed as biosensors in bioimaging, drug delivery, and in the photoreduction of metals, since they have electron transfer and redox properties. There are two main methods to synthesize CNDs: top-down (e.g., laser ablation, electrochemical synthesis) and bottom-up (e.g., combustion, microwave irradiation) [1–2]. In particular, the use of microwave (MW) irradiation is an interesting synthetic approach, which allows several molecular precursors to be employed, such as amino acids in aqueous solution [3]. As an example, L-tyrosine was used to form hydrophobic CNDs able to sense ions and silver nanoparticles [4]. Arginine or cysteine have also been efficiently employed as starting materials through a hydrothermal route [5]. Alternatively, in a convenient MW-based method, arginine was shown to be a useful starting material towards highly fluorescent nitrogen-doped CNDs (NCNDs) that were chosen for the present study (Scheme 1) [6].

Several works have reported incorporation of CNDs into hydrogels as an interesting method to control fluorescence quenching upon application of a specific trigger, and to introduce new physical and optical properties of interest [7–8]. Such systems can be useful in several applications, such as bacteria detection [9], sensing of reactive oxygen species (ROS) and screening for apoptotic activity of 5-fluorouracil [10]. Hydrogels containing CNDs reported thus far are mainly composed of cross-linked macro-polymer networks, such as poly-(*N*-vinylcaprolactam) (PVCL) [11] or poly(*N*-isopropylacrylamide) (PNIPAM), which is a thermoresponsive polymer suitable for biomedical and environmental applications [12–13]. Carbohydrates such as chitosan [14–15], alginate [16], and agarose [7,17] hydrogels have also been used to incorporate CNDs.

Only a very few studies have incorporated CNDs into supramolecular hydrogels obtained from low-molecular-weight gelators (LMWGs). Relative to gelling polymers, LMWGs have many advantages including well-defined chemical composition, and the possibility to achieve reversible gelation upon application of a specific trigger. Thus, smart, soft materials that can adapt to the environment can be obtained, mimicking natural biological tissues to address demanding therapeutic challenges [18]. In 2015, Steed et al. reported CND–hydrogel hybrids obtained from bis(urea) derivatives used as LMWGs [19] that displayed considerable fluorescence enhancement relative to CNDs alone and showed promising performance in silver ion selective determination [20]. In 2016, the interesting hydrogelation ability of guanosine 5′-monophosphate (5′-GMP)-derived CNDs was also reported [21]. To the best of our knowledge, the addition of CNDs to peptide-based hydrogels has not yet been investigated, despite this being an interesting class of supramolecular soft materials.

Peptide self-assembled hydrogels are inherently biocompatible and biodegradable and thus are promising biomaterials for cell culture, regenerative medicine, tissue engineering, and drug delivery applications [22]. The identification of self-assembling peptides that are as short as possible is highly useful due to the low cost and simplicity of synthesis, as opposed to longer peptides that require solid-phase-peptide synthesis [23]. The most typical approach employs *N*-capped short peptides, especially whereby the *N*-capping group is a hydrophobic, aromatic moiety that assists self-assembly in water [24]. In 2012, the first systems of uncapped tripeptides were reported to self-assemble into nanostructured hydrogels at physiological conditions and without the need for organic solvents. These tripeptides were heterochiral, that is, composed of both D- and L-amino acids, and they formed hydrogels following a pH change, while their homochiral stereoisomers did not.

In particular, the tripeptide ^D^Leu-Phe-Phe, which was chosen for the present study, immediately formed a self-supporting hydrogel [25]. In a typical protocol, the tripeptide was first dissolved as an anion in an alkaline buffer thanks to electrostatic repulsion between molecules. Then, the addition of a second buffer was used to lower the pH to neutral. This tripeptide proved to be a strong gelator able to co-assemble into nanostructured hydrogels with aromatic small molecules. In this manner, it yielded a useful vehicle for the sustained release of the poorly soluble antibiotic ciprofloxacin [26]. Fluorescent hydrogels were formed from co-assembly with a dye into nanostructures of different morphology, depending on whether the dye was added initially to the peptide in the alkaline buffer solution, or later to the second buffer that triggered self-assembly, thus showing different outcomes depending on the protocol used [27].

In this study, we report for the first time two different protocols for NCND incorporation into supramolecular hydrogels composed of an uncapped tripeptide, ^D^Leu-Phe-Phe, and characterize the system by rheometry, fluorescence, circular dichroism (CD), FTIR spectroscopy, transmission electron microscopy (TEM), and differential scanning calorimetry (DSC). Given that this tripeptide is capable of forming a hydrogel with mild antimicrobial activity and a lack of cytotoxicity in vitro [26], this new system could be valuable for the development of wound healing applications [28], whereby luminescence could be advantageous to visually track the presence of the hydrogel and the ability of its components to penetrate through the derma [29]. In addition, peptide hydrogels based on the Phe-Phe motif [30–31] and bearing unnatural D-amino acids [32–33] are attractive biomaterials that may display higher durability relative to traditional peptide counterparts, in addition to better biocompatibility and the possibility to incorporate bioactive motifs relative to non-peptide hydrogels. Therefore, in the long term, a supramolecular hydrogel composed of a peptide and luminescent nanodots could be valuable for tissue regeneration based on a bioactive scaffold that can be also visualized in vivo by fluorescence microscopy. Alternatively, other potential applications could be developed in the future for drug delivery and even sensing, if the nanodots were suitably derivatized to release a drug or undergo fluorescence quenching upon binding of a specific target molecule.

## Results and Discussion

### Peptide self-assembly in the presence of NCNDs

The incorporation of carbon nanostructures into hydrogels is a useful approach to introduce additional properties to soft materials. In the case of self-assembling peptides, non-covalent π–π interactions between the nanocarbon and aromatic residues of the peptide offer a convenient means to bring the two components together into a supramolecular system [34]. This rationale could also be applied to NCNDs and the tripeptide ^D^Leu-Phe-Phe, which were evaluated for co-assembly into hydrogels following a pH trigger from alkaline to neutral. Different scenarios were envisaged: the presence of the NCNDs 1) could promote peptide self-assembly by acting as a nucleation agent, 2) could hinder self-assembly of the peptide, or 3) might not interact with the peptide. To verify the effects of NCNDs on the supramolecular behavior of the tripeptide, a series of experiments were performed as outlined in Table 1, with different amounts of each component dissolved in either buffer or together in the alkaline buffer.

**Table 1:** Experiments to probe the effects of NCND presence on peptide self-assembly (SA).

Peptide final concentration^a^	NCND concentration (relative to the peptide)	Hydrogel formation? NCND prior to SA^b^	Hydrogel formation? NCND during SA^c^

5 mM	0.02 mg/mL (1% w/w)	No	Yes
5 mM	≥0.05 mg/mL (2.5% w/w)	No	No
10 mM	0.04 mg/mL (1% w/w)	No	Yes
10 mM	0.1 mg/mL (2.5% w/w)	No	Yes
10 mM	0.2 mg/mL (5% w/w)	No	Yes
10 mM	≥0.4 mg/mL (10% w/w)	No	No
15 mM	0.7 mg/mL (10% w/w)	Yes	Yes
**15 mM**	**1.0 mg/mL (15% w/w)**	**Yes**	**Yes**
15 mM	≥1.4 mg/mL (20% w/w)	No	No

^a^The peptide alone forms hydrogels already at 5 mM. ^b^NCNDs are dispersed in the alkaline buffer. ^c^NCNDs are dispersed in the acidic buffer.

**Figure 1:**
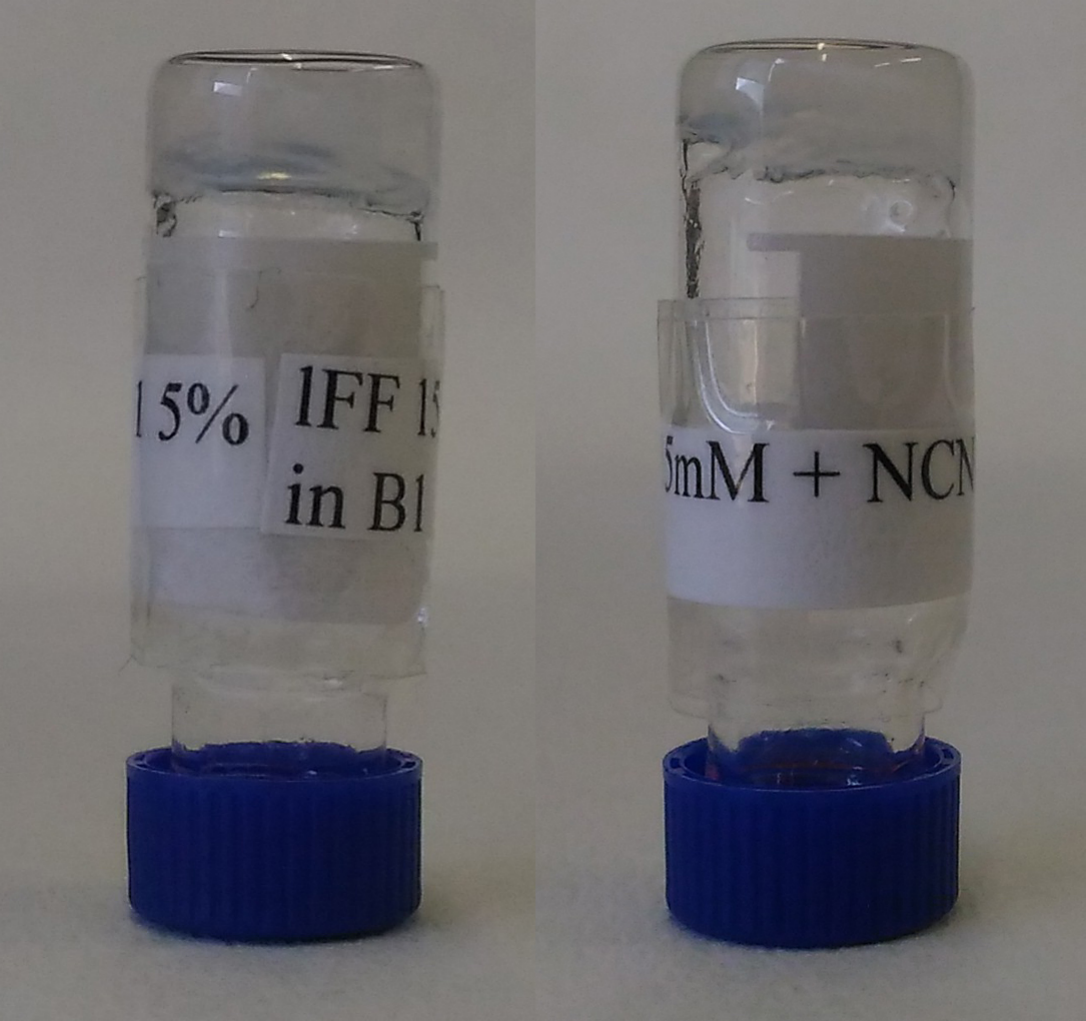
Hydrogels obtained from ^D^Leu-Phe-Phe (15 mM) and NCNDs at 1 mg/mL (15% w/w relative to the peptide) dissolved either in alkaline buffer with the peptide prior to self-assembly (left), or in acidic buffer that was added to the peptide alkaline solution to trigger self-assembly (right).

It is apparent that the presence of NCND hindered peptide supramolecular organization, and even more so when peptide and NCND were dissolved together prior to self-assembly (i.e., NCND and peptide were both added to the alkaline buffer). Increasing the peptide concentration up to nearly its solubility limit (i.e., for a final peptide concentration of 15 mM in the hydrogel) progressively increased the amount of NCND that could be tolerated by the peptide to achieve self-assembly, up to a maximum of 1 mg/mL or 15% w/w relative to ^D^Leu-Phe-Phe (highlighted in Table 1). In this case, self-supportive hydrogels were formed regardless of the protocol used (i.e., addition of the NCNDs to either alkaline or acidic buffer), as shown in Figure 1. Both conditions were further investigated since peptide nanostructure morphology may change significantly upon co-assembly with other molecular components, depending onto whether the latter were added either to the alkaline or the acidic buffer [27].

### Rheological properties of NCND–peptide hydrogels

**Figure 2:**
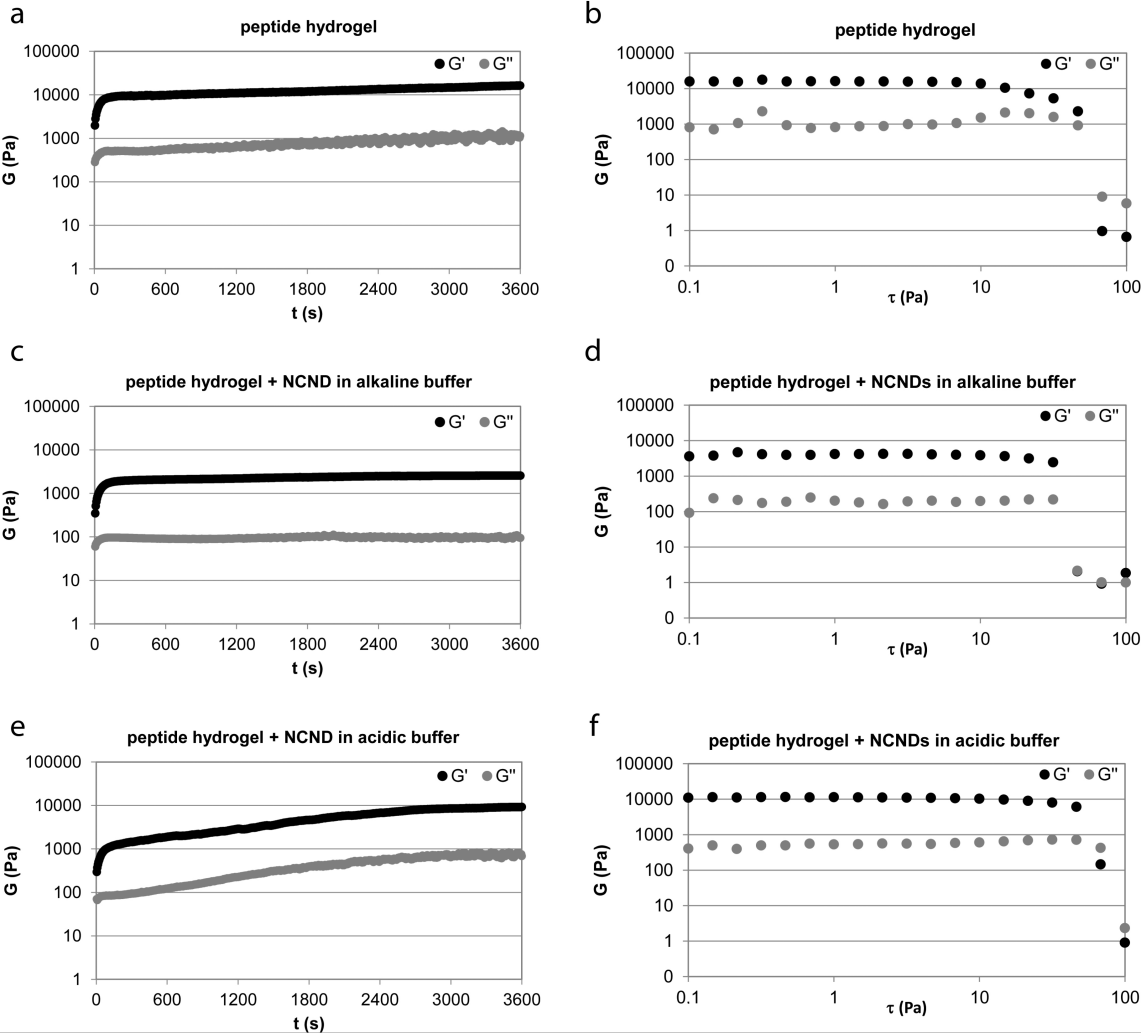
Oscillatory rheometric data for the hydrogels. Time sweeps (left) and stress sweeps (right) for the hydrogel composed of the peptide alone (a,b), and with NCNDs added either to alkaline buffer (c,d) or to acidic buffer (e,f).

The rheological properties of the hydrogels were assessed by means of oscillatory rheometry (Figure 2). In all cases, gelation was so rapid that the monitoring of the sol-to-gel transition was not possible. Time sweep experiments (Figure 2a,c,e) revealed that relative to the peptide alone, which reached an elastic modulus G’ of 20 kPa within 1 h (Figure 2a), the addition of NCNDs to the peptide prior to self-assembly (Figure 2c) did not slow down gelation kinetics. Both the elastic (G’) and viscous (G’’) moduli were significantly reduced, yielding softer hydrogels (G’ of 3 kPa within 1 h). Instead, when the NCNDs were added to the peptide during the pH trigger, gelation kinetics were slowed down. However, over 1 h, the hydrogel had already reached an elastic modulus of 10 kPa, thus yielding a stiffer material relative to the former case (Figure 2e). In any case, at any given time point, the hydrogels containing NCNDs displayed a lower elastic modulus G’ relative to the peptide alone. This phenomenon could be compatible with the presence of thinner bundles of fibers.

Stress sweeps (Figure 2b,d,f) were employed to monitor variations in the hydrogel resistance to applied stress. Relative to the peptide alone (Figure 2b), NCND addition (Figure 2d,f) increased the linear viscoelastic region, thus improving the material stability to external forces, especially when NCNDs were added to the acidic buffer. This observation was compatible with better interconnected networks of fibrils in the presence of NCND. Frequency sweep experiments confirmed in all cases a hydrogel nature with G’ > G’’ and both G’ and G’’ independent of the applied frequency (see Supporting Information File 1).

Overall, from a rheological point of view, the addition of NCNDs increased the peptide hydrogel stability to applied stress and offered the opportunity to fine-tune stiffness or gelation kinetics, depending on the protocol used to prepare the material.

### Fluorescence properties of NCND–peptide hydrogels

**Figure 3:**
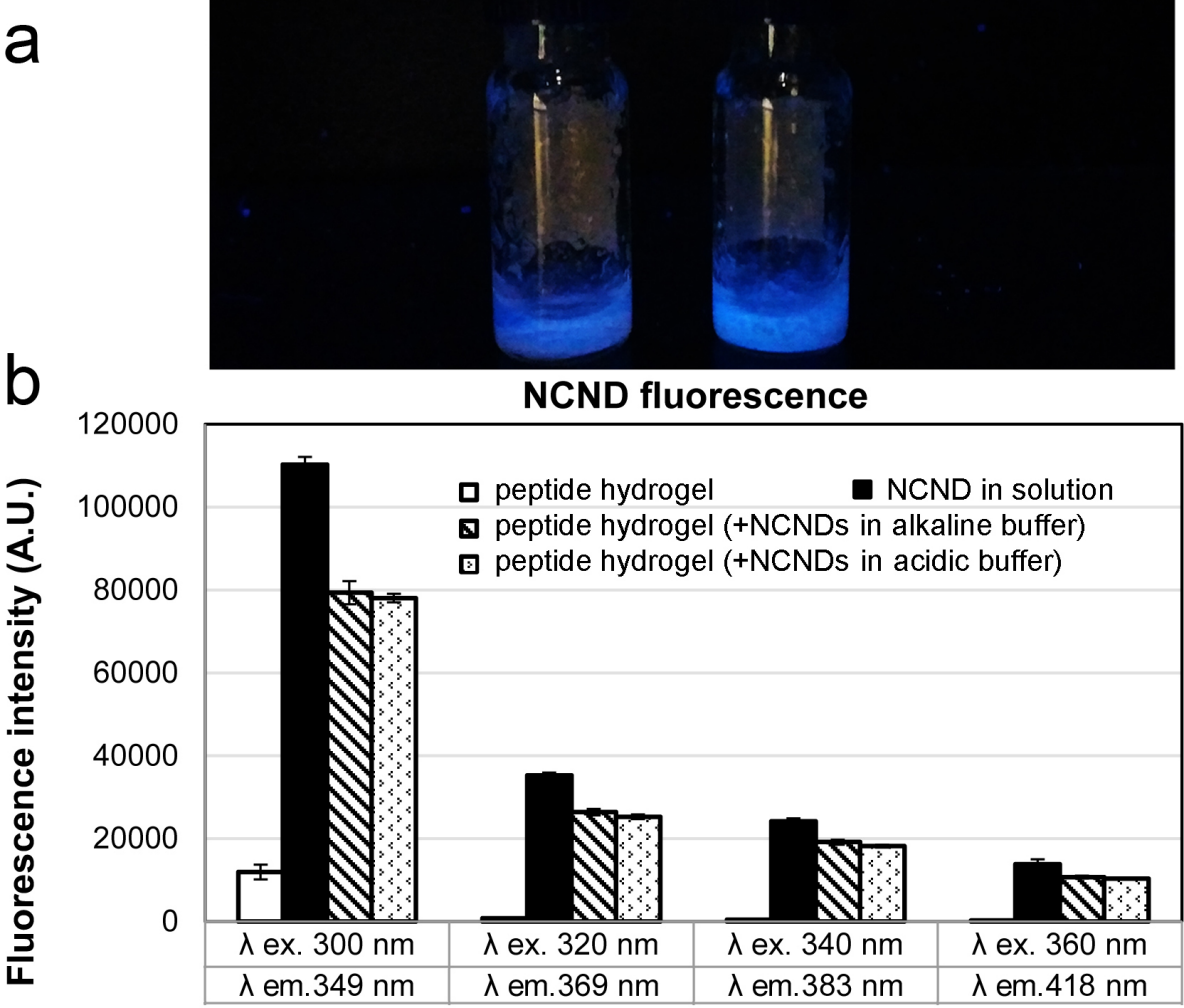
a) NCND–peptide hydrogel fluorescence as seen under UV-light illumination. b) Excitation-dependent fluorescence of the hydrogels and NCNDs in solution.

The excitation-dependent fluorescence emission range of the NCNDs was probed within the hydrogel structure (Figure 3). As expected, the peptide hydrogel showed negligible fluorescence properties at the wavelengths explored, while the NCNDs displayed intense fluorescence, especially in the UV region [6]. Relative to NCNDs in solution, their incorporation within the hydrogel matrix resulted in a decrease of their fluorescence emission intensity, accounting for nearly 30% upon excitation at 300 nm, and 20–25% upon excitation at longer wavelengths. Nevertheless, the hydrogels were intensely luminescent as seen under UV-light illumination (Figure 3a). No significant difference was observed between the materials prepared according to the two different protocols. Importantly, upon incorporation into the hydrogel matrix, no shift in NCND fluorescence emission spectra was registered, and the NCND fluorescence stability was not affected over a 7-day period.

### Peptide conformation in the presence of NCNDs

**Figure 4:**
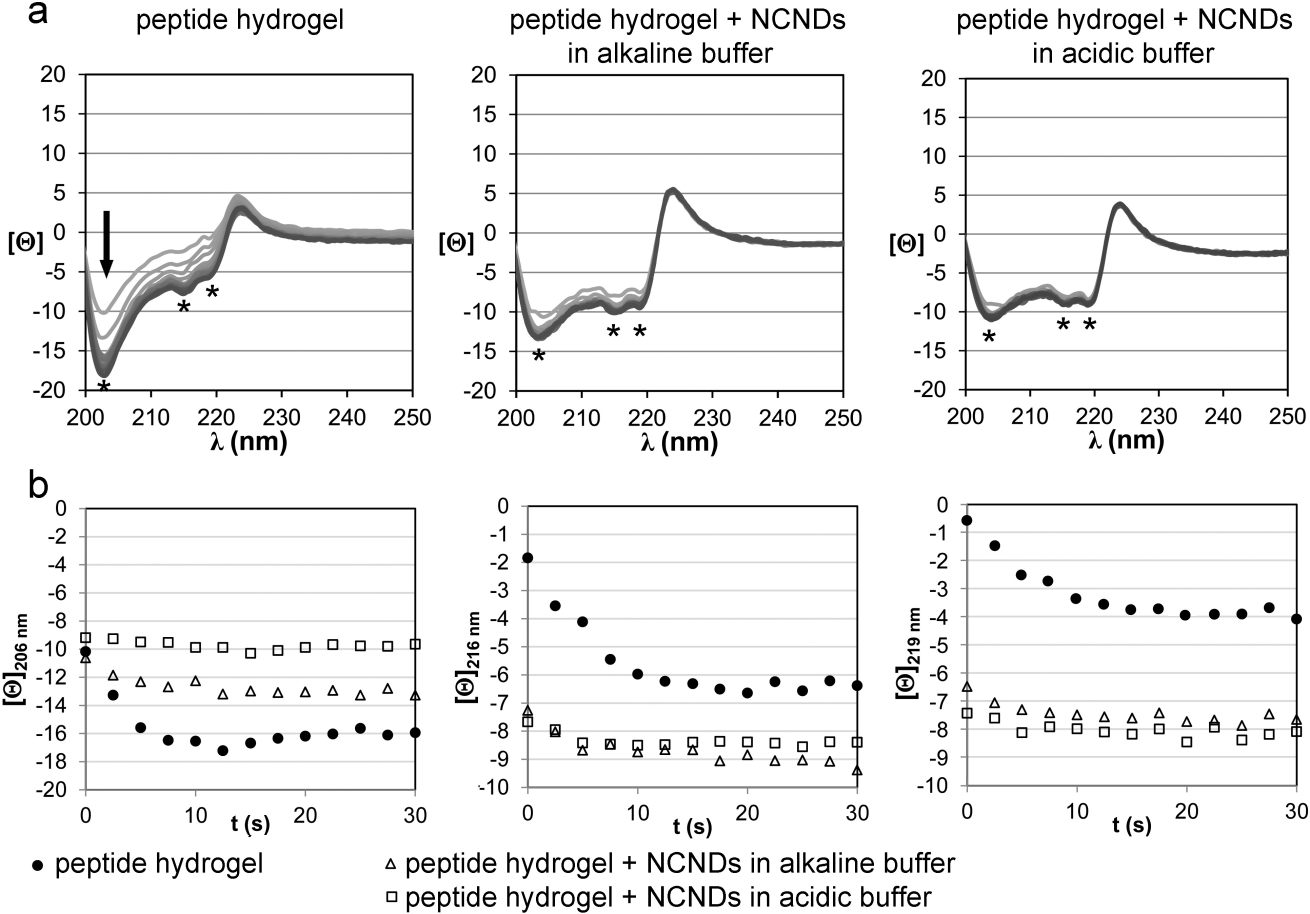
a) CD spectra of self-assembled hydrogel evolution over time. The arrow indicates the direction of signal evolution. The amide signals display three minima at 206, 216 and 219 nm (denoted by *) that are displayed individually over time in b). Note: plotted [Θ] units have been divided by 1000.

Peptide conformation was assessed by means of circular dichroism (CD), FTIR spectroscopy, and thioflavin T fluorescence. CD was used to monitor self-assembly over one hour (Figure 4). In all cases, self-assembly led to a spectrum that was very distinctive of the supramolecular structure and markedly different to the peptide in solution (see Supporting Information File 1). Overall, the main features of the peptide hydrogel CD spectra were maintained after NCND addition with quantitative rather than qualitative differences observed. Signal evolution occurred mainly during the initial 10 minutes, with only minor variations over time for the NCND-containing hydrogels.

The 200–220 nm region, which is attributed to amide signals and is thus related to peptide conformation, was characterized by negative minima that were compatible with supramolecular beta-sheets. In particular, three minima were present: one at 206 nm that was more intense for the peptide hydrogel, and another two at 216 and 219 nm that were more intense after addition of NCNDs. In particular, the intensity of the latter was nearly doubled in the presence of NCNDs (Figure 4b). FTIR spectroscopy did not reveal significant differences in the amide I signal between samples (see Supporting Information File 1), suggesting that NCND addition did not significantly affect overall peptide conformation (e.g., from beta-sheets to random coil or else). This hypothesis was further supported by the CD spectrum of the peptide in solution that was unchanged in the presence of NCNDs (see Supporting Information File 1).

Thioflavin T fluorescence was thus used to further understand NCND effects on the peptide supramolecular structure. Thioflavin T is a dye that binds to hydrophobic grooves formed by at least four consecutive beta-strands, leading to fluorescence that is used to assess the peptide amyloid character [35]. Fluorescence arises from the limited rotation of a single bond between two aromatic rings composing the dye, namely the benzothiazole and the dimethylanilino units [36]. Although its fluorescence can also be increased by an increase of solvent viscosity [36], in aqueous environments, it is effectively and universally used as an amyloid marker thanks to its ability to laterally bind to the surface of peptide fibrils [37]. This interaction has been the subject of numerous studies that overall elucidated that an increase in fluorescence intensity linearly correlates to amyloid fibril concentration [38].

**Figure 5:**
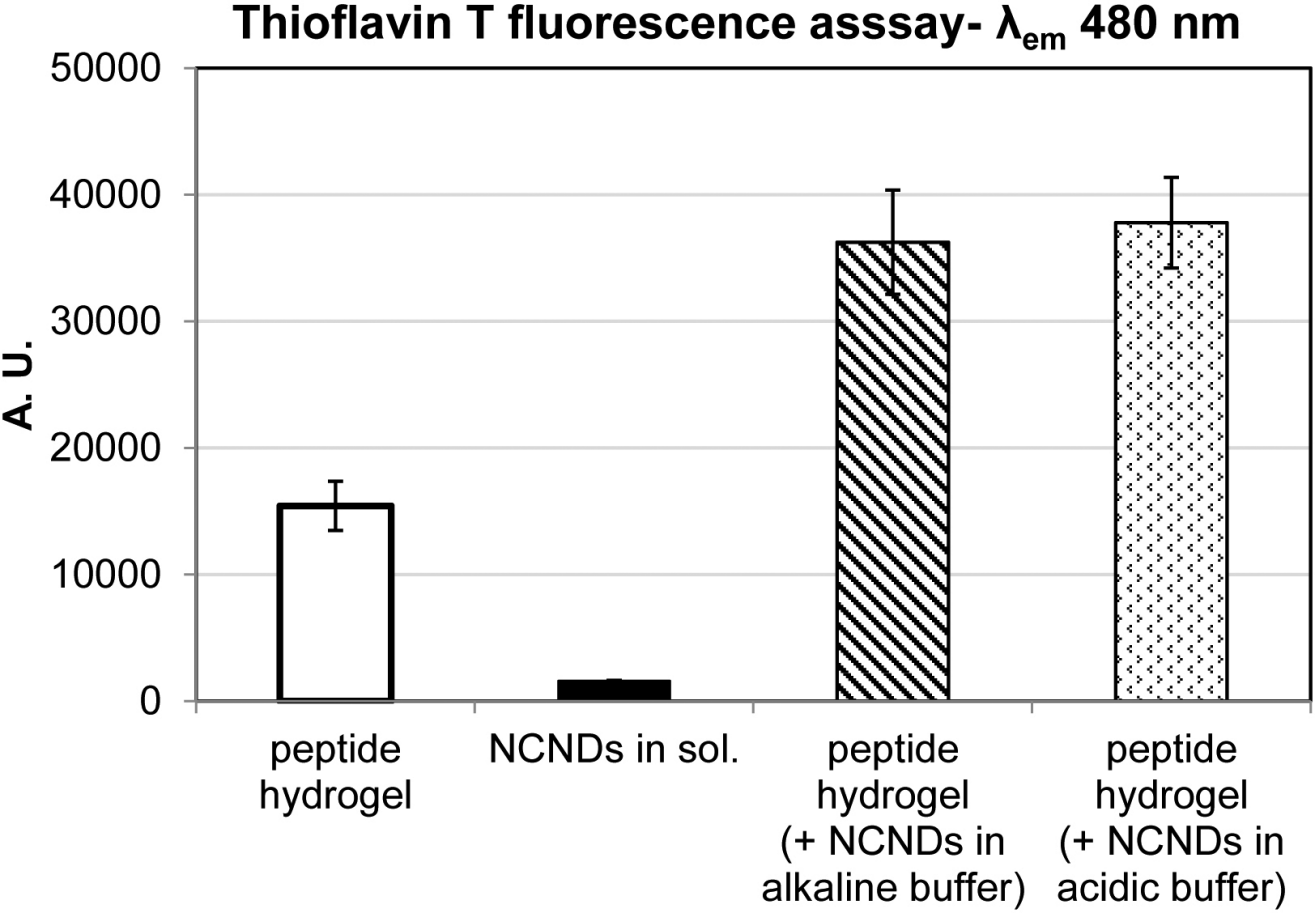
Thioflavin T fluorescence assay.

In the presence of the dye, the NCNDs showed negligible fluorescence at the wavelength probed, in contrast with the self-assembled peptide, which is in agreement with the literature [25]. Unexpectedly, the addition of NCNDs to the hydrogel led to an over a two-fold increase in thioflavin T fluorescence, regardless of the protocol used (Figure 5). Considering that the addition of NCNDs reduced the overall viscosity of the hydrogel systems, as revealed by rheometry, it is unlikely that the noted increase in fluorescence is to be ascribed to viscosity variations. Overall, while NCNDs did not modify peptide conformation, they appeared to favor the formation of supramolecular extended beta-sheets that could bind thioflavin T. This resulted in more intense CD minima at 216 and 219 nm and more intense thioflavin T fluorescence.

### Nanostructure morphology of NCND–peptide hydrogels

**Figure 6:**
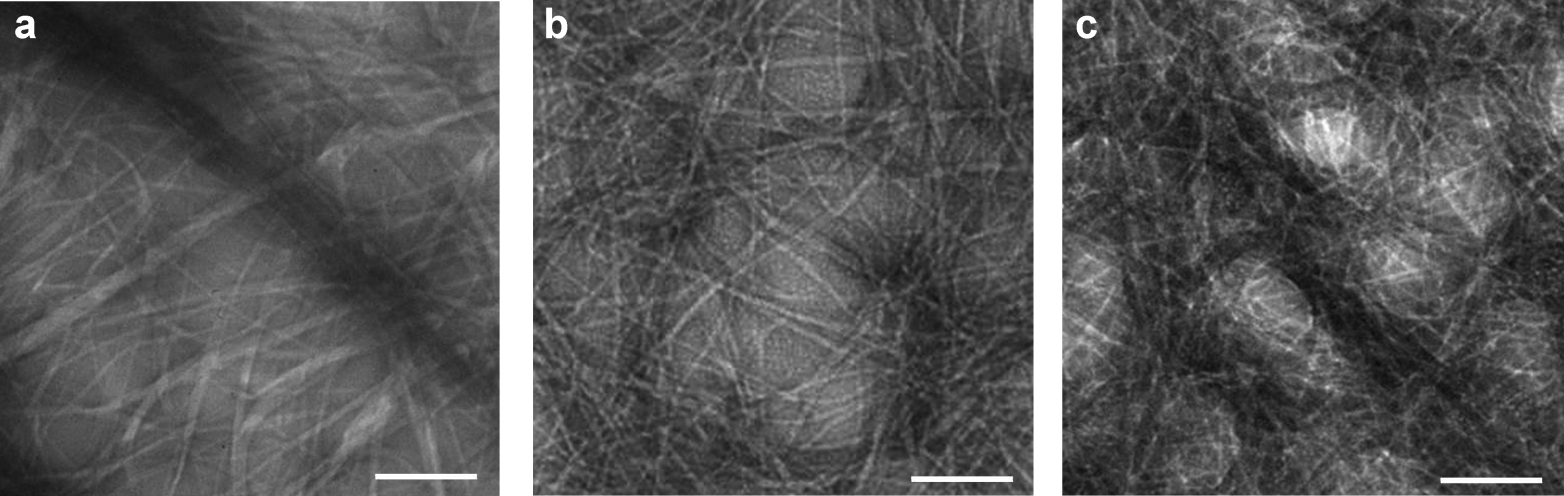
TEM micrographs of hydrogels containing peptide (a) and NCNDs added to either alkaline (b) or acidic (c) buffer. Scale bar = 200 nm for all images.

Transmission electron microscopy (TEM) was used to assess the nanostructure of the hydrogels (Figure 6). Once self-assembled, the tripeptide formed elongated fibrils that bundled into thicker fibers, forming a three-dimensional network that entrapped water. Typical hydrogel samples composed of peptide alone under TEM imaging appeared as networks of fibers of highly heterogeneous thickness, with a wide distribution ranging from individual fibrils to thick bundles that grow in thickness over time [25]. The TEM imaging performed in the present study confirmed the presence of the anisotropic structures in all cases, with no significant difference in individual fibril diameter upon addition of NCNDs (i.e., 9 ± 3 nm for the peptide alone, and 10 ± 2 nm upon addition of NCNDs, regardless of the protocol used). In all cases, there was a high density of fibrils with length exceeding the field-of-view of several micrometers, thus hindering the possibility to quantify minor differences in fibril number or length. However, the number of fibrils running in parallel, reflecting their tendency to bundle, appeared higher in the absence of NCNDs, which may play a role in explaining the thioflavin T fluorescence data. Indeed, the presence of higher numbers of thinner and less bundled fibrils could result in a higher accessible surface area for thioflavin T binding.

Due to their small diameter (<2 nm), the NCNDs could not clearly be discerned individually by TEM. However, their presence was compatible with less dense areas in between fibrils, in agreement with a lack of a strong interaction of the peptide. However, it was not possible to identify whether NCNDs were also present within the peptide fibrils, giving scope for future investigations to elucidate these systems in further detail.

In a separate set of experiments, a gradual pH change was also investigated to monitor the effects on nanofibril morphology. Briefly, the samples were prepared as previously described, but the acidic buffer was added dropwise to achieve the desired pH. During sample preparation, it was evident that as soon as each drop of the second buffer was added to the system, peptide self-assembly immediately occurred locally before the system could be homogenized by mixing. As a result, all samples displayed a heterogeneous nature, with gel mass and liquid phase around it until gelation was complete. Such a heterogeneous nature was also present at the nanoscale, as revealed by TEM, with wide distribution of fiber diameters in all samples, with and without NCNDs (see Supporting Information File 1). These observations are not surprising since it is well known that the nanostructure outcome of self-assembly is greatly influenced by experimental conditions and gelation kinetics [39–40].

### Thermal stability of NCND–peptide hydrogels

The NCND–peptide hydrogels were assessed for their thermal stability by means of differential scanning calorimetry (DSC) and circular dichroism (CD) with a heating ramp from room temperature up to complete disappearance of the CD signal (see Supporting Information File 1).

DSC did not reveal major differences in the gel-to-sol transition temperature amongst samples. In all cases a first, a wide and asymmetric endotherm was observed, whose minimum relative to *T*_m_ was displayed at approximately 77–82 °C. A second, narrow endotherm with a minimum just above 100 °C could be ascribed to the evaporation of residual buffer solution. It is worth noting that the DSC data of similar peptides and amyloids often display multiple minima that are not always discernible and result in wide, asymmetric endotherms; besides the gel-to-sol transition, other minima in the range of 80–85 °C can be ascribed to aggregates formed during heating [41–42]. It is thus possible that the wide endotherm observed in this work is the sum of all such different components. As a result, minor differences in the thermal stability of the systems with or without NCNDs may have been masked. Indeed, the small DSC sample volumes include as little as 19 µg of NCNDs.

For this reason, we next performed CD with a heating ramp until complete disappearance of the UV signal that monitors specifically peptide conformation and the resulting supramolecular chiral environment. The peptide hydrogel samples displayed progressive reduction of the CD signal until 80 °C, in agreement with DSC data. The samples containing NCNDs in either buffer displayed an anticipated loss of the supramolecular chiral environment that was complete at 70 °C (see Supporting Information File 1). Such reduction in thermal stability is compatible with the thinner fibers observed by TEM upon addition of NCNDs. A minor discrepancy between the absolute values obtained with the two techniques could also be ascribed to sample holder geometries that differ in their surface-to-volume ratios (which is much higher in the CD cell), as well as different heat transfer systems for the two instruments.

## Conclusion

For the first time we reported herein two convenient protocols for the rapid preparation of luminescent supramolecular hydrogels formed by a tripeptide in the presence of NCNDs at physiological conditions. It was shown that relative amounts of peptide and NCNDs needed optimization to allow self-assembly and gelation, which occurred with up to 15% w/w of NCNDs relative to the peptide. Nevertheless, rheometric analyses revealed that NCNDs increased the linear viscoelastic region of the hydrogel, thus resulting in increased stability of the soft material to applied stress. Importantly, the addition of NCNDs offered the opportunity to fine-tune the gelation kinetics as well as the stiffness of the final material, thus opening new windows of use depending on the intended final application.

Interestingly, neither the beta-sheet peptide conformation nor the individual fibril nanostructure in the hydrogel were significantly changed by the presence of NCNDs. However, both circular dichroism and thioflavin T fluorescence revealed signs of interaction between the two components at the supramolecular level that were compatible with increased concentration of thinner fibres, as opposed to thick bundles, with higher surface area available for thioflavin T binding. As a result, the hydrogels containing NCNDs displayed a narrower fiber diameter distribution with overall thinner structures that were better interconnected, which was in agreement with the rheological observations discussed above. Importantly, NCND addition not only provided luminescence to the hydrogels, but also allowed control over the well-known issue of heterogeneous thickness of supramolecular peptide fibers, resulting in improved viscoelastic properties of the final materials.

## Experimental

All chemicals were purchased from Sigma-Aldrich. All solvents were from Merck. High purity Milli-Q water (MQ water) with a resistivity greater than 18 MΩ·cm was obtained from an in-line Millipore RiOs/Origin system.

### Synthesis and characterization

The tripeptide ^D^Leu-Phe-Phe was synthesized according standard Fmoc solid phase peptide synthesis and purified by RP-HPLC, as previously described [25]. The peptide identity and purity was verified by ESI–MS, ^1^H NMR and ^13^C NMR. The as-produced NCNDs were synthesized and purified following a reported procedure [6].

### Sample preparation

Tripeptide hydrogels were prepared in phosphate buffer as previously described [25] at the desired concentration as described in Table 1. Briefly, the peptide was dissolved in a 0.1 M solution of sodium phosphate at pH 11.8 (alkaline buffer), and then an equal volume of 0.1 M solution of sodium phosphate buffer at pH 5.8 (acidic buffer) was added to reach a final pH of 7.3 ± 0.1, as verified with a pH meter. For the preparation of the peptide hydrogels containing NCNDs, NCNDs were dispersed either in the alkaline buffer (with the peptide) or in the acidic buffer, at various concentrations as described in Table 1.

### Rheometry

The dynamic time sweep rheological analysis was conducted on a Malvern Kinexus Ultra Plus rheometer with a 20 mm stainless steel parallel plate geometry. The temperature was maintained at 25 °C using a Peltier temperature controller. The samples were prepared in situ and immediately analyzed with a gap of 1.00 mm. Time sweeps were recorded for 1 h using a frequency of 1.00 Hz and a controlled stress of 5.00 Pa. After 1 h, the frequency sweeps were recorded using a controlled stress of 5.00 Pa and then stress sweeps were recorded using a frequency of 1 Hz.

### Circular dichroism (CD) spectroscopy

A 0.1 mm quartz cell was used on a Jasco J815 spectropolarimeter, with 1 s integration time, 1 accumulation, and a step size of 1 nm with a bandwidth of 1 nm over a wavelength range of 200–250 nm. The samples were freshly prepared directly in the CD cell and the spectra were immediately recorded. The spectra were recorded at 25 °C or with a heating ramp up to 80 °C and 5 °C steps. The control samples with only NCNDs in buffer solutions (without peptide) did not show any signal in the region analyzed.

### Fluorescence assay

Gel precursor solutions were prepared as described above and 100 μL of each buffer were immediately put on wells of Greiner 96 U Bottom Black Polystyrene. The controls were used in 200 μL total volume. After 1 h, the fluorescence emission spectra were acquired using a Tecan Infinite M1000 pro, with a bandwidth of 10 nm, selecting the following excitation (ex.) and emission (em.) wavelengths: ex. 300 nm and em. 325–499 nm (maximum at 349 nm); ex. 320 nm and em. 345–499 nm (maximum at 369 nm); ex. 340 nm and em. 365–499 nm (maximum at 383 nm); ex. 360 nm and em. 385–520 nm (maximum at 418 nm). Each condition was repeated at least twice in triplicate. The average and standard deviations were calculated and plotted.

### Thioflavin T fluorescence assay

Gel precursor solutions were prepared as described above and 100 μL of each buffer were immediately put on wells of Greiner 96 U Bottom Black Polystyrene. The controls were used in 200 μL total volume. After 1 h, 20 μL of a solution of thioflavin T (22.2 μM in 20 mM glycine/NaOH pH 7.5, filtered with a 0.2 μm filter) were added in the wells. After 15 min, the fluorescence emission was analyzed using a Tecan Infinite M1000 pro, selecting an excitation wavelength of 446 nm and an emission wavelength range from 470 to 560 nm, with a bandwidth of 10 nm. Each condition was repeated at least twice in triplicate. The average and standard deviations were calculated and plotted.

### TEM imaging

TEM micrographs were acquired on a Jeol, JEM 2100 instrumen (Japan) at 100 kV. TEM grids (copper-grid-supported lacey carbon film) were first exposed to a UV-ozone cleaner (UV-Ozone Procleaner Plus) for 45 mins to make the grid surface more hydrophilic. Then, the six-hour-aged gels were precisely deposited on a TEM grid, dried for 15 min at room temperature, and contrasted by an aqueous tungsten phosphate solution (pH 7.4). The average size or cross-section diameter of the nanostructures was determined by taking into account at least 100 individual nanostructures.

## Supporting Information

The supporting information includes FTIR methods and spectra, additional rheometry and CD data, DSC data, and additional TEM images for the gradual pH change experiments.

File 1 Additional experimental information.
