# Supplementary material for: Luminescent supramolecular hydrogels from a tripeptide and nitrogen-doped carbon nanodots
Source: Beilstein J Nanotechnol. 2017 Aug 1;8:1553–62. doi: 10.3762/bjnano.8.157 (PMC5550813; doi:10.3762/bjnano.8.157)

Supporting Information

for

Luminescent supramolecular hydrogels from a tripeptide and nitrogen-doped carbon nanodots

Maria C. Cringoli[1], Slavko Kralj[1,2], Marina Kurbasic[1], Massimo Urban[1], and Silvia Marchesan*[1]

Address: [1]Department of Chemical and Pharmaceutical Sciences, University of Trieste, Via L. Giorgieri 1, Trieste 34127, Italy, and [2]Department for Materials Synthesis, Jožef Stefan Institute, Jamova 39, Ljubljana 1000, Slovenia

Email: Silvia Marchesan - smarchesan@units.it

* Corresponding author

Additional Experimental Information

Peptide spectroscopic data

^{1}H-NMR (500 MHz, DMSO):δ (ppm) 8.73 ppm (d, *J* = 8 Hz, 1H, NH), 8.58 ppm (d, *J* = 8 Hz, 1H, NH), 7.48-7.03 ppm(m, 10H, Ar), 4.77-4.62 ppm (m, 1H, αCH), 4.47 ppm (m, 1H, αCH), 3.63 ppm (m, 1H, αCH), 3.18-3.00 ppm (m, 2H, βCH), 2.96 ppm (dd, 1H, *J* = 14.0; 9.1 Hz, βCH), 2.65 ppm (dd, *J* = 13.7; 11.5 Hz, 1H, βCH), 1.19-1.08_ ppm (m, 3H, β and γ CH), 0.66 ppm (dd, 6H, δCH). ^{13}C-NMR (125 MHz, DMSO): δ (ppm) 173.2, 171.6, 169.2 (CO); 138.0, 137.9, 129.8, 129.6, 128.7, 128.4, 127.0, 126.8 (Ar); 54.2, 54.1, 51.0, (αC); 40.7, 38.6, 37.0 (βC); 23.6 (γC) 22.9, 22.2 (δC). MS (ESI): m/z 426.2 (M+H), $C_{24}H_{31}N_3O_4$ requires 425.2.

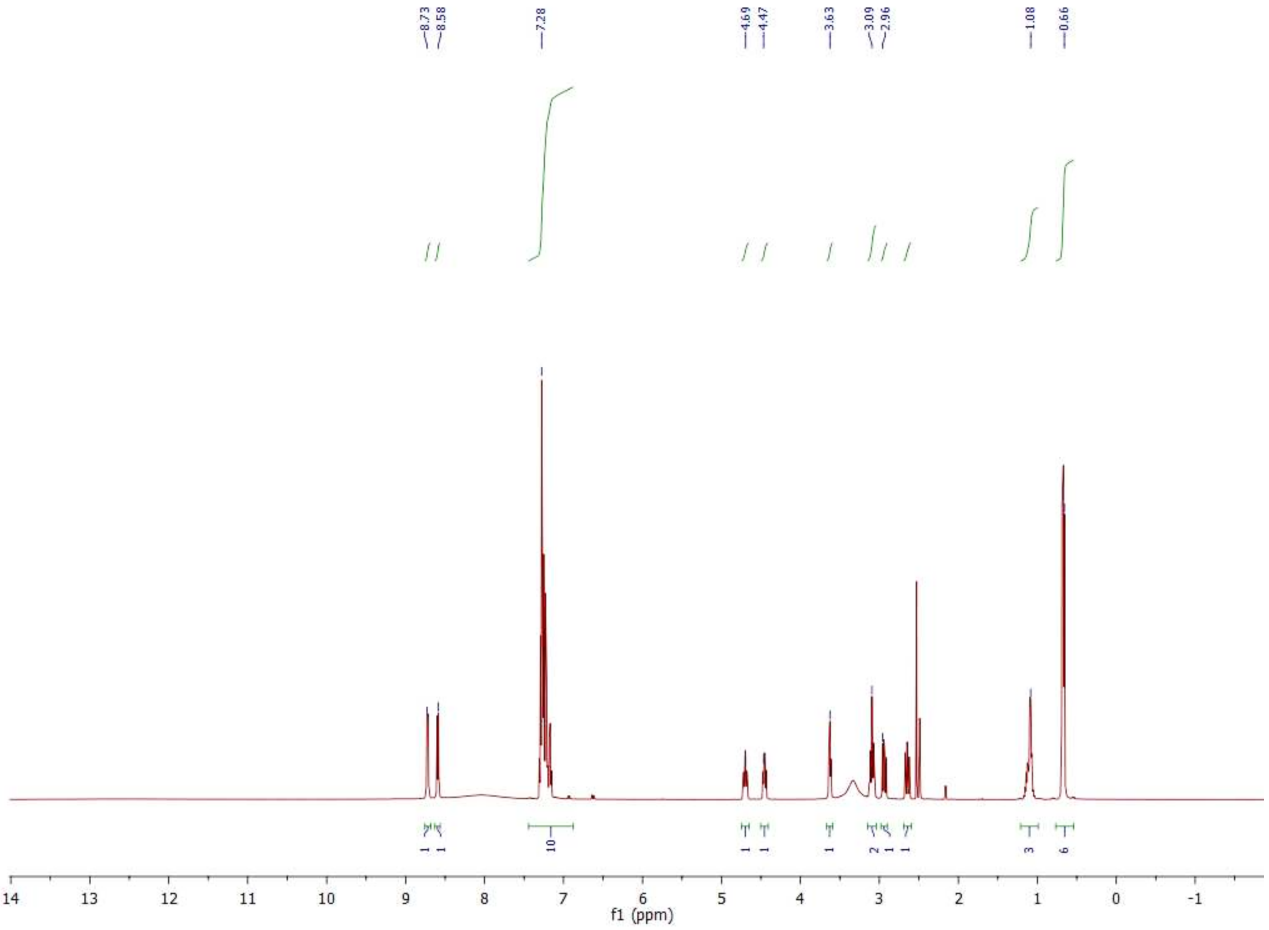

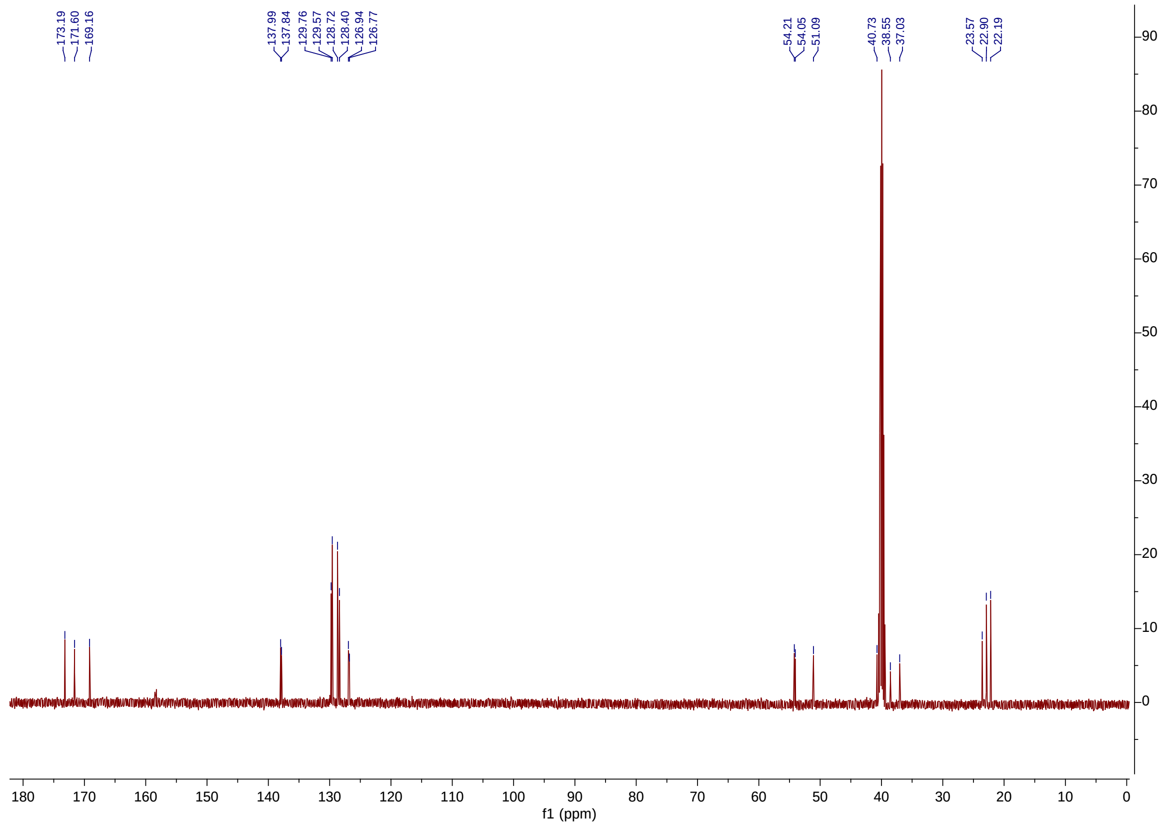
173.19
171.60
169.16
137.99
137.84
129.76
129.57
128.72
128.40
126.94
126.77
54.21
54.05
51.09
40.73
38.55
37.03
23.57
22.90
22.19
f1 (ppm)

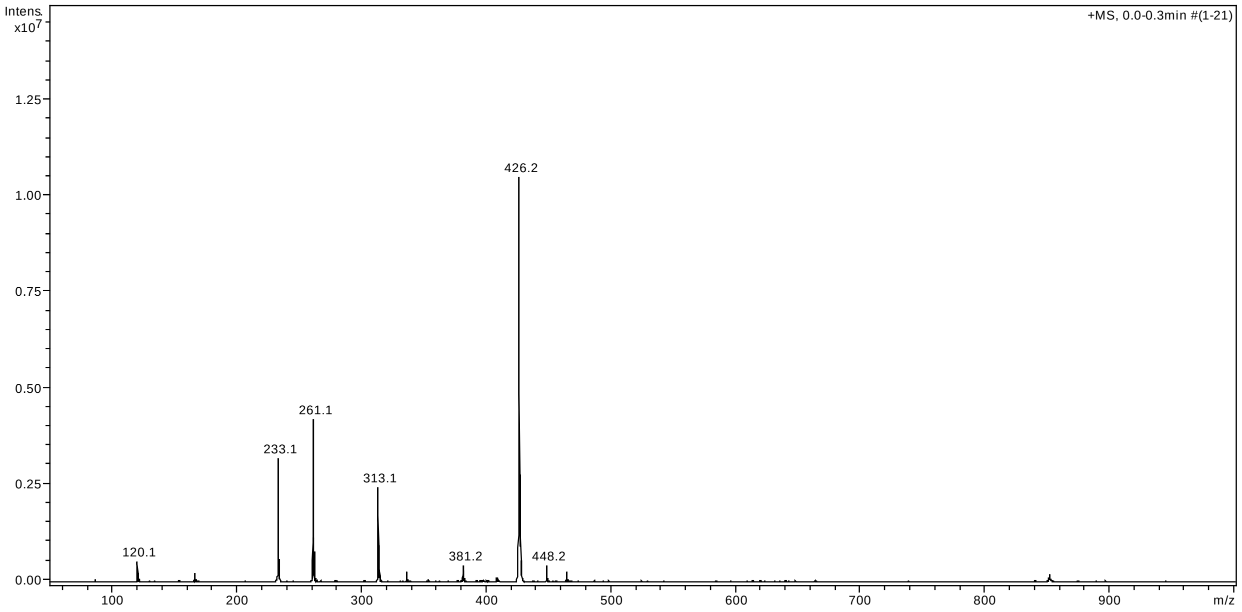
Intens.
x10^7
+MS, 0.0-0.3min #(1-21)
426.2
261.1
233.1
313.1
120.1
381.2
448.2
m/z

Rheometry frequency sweeps

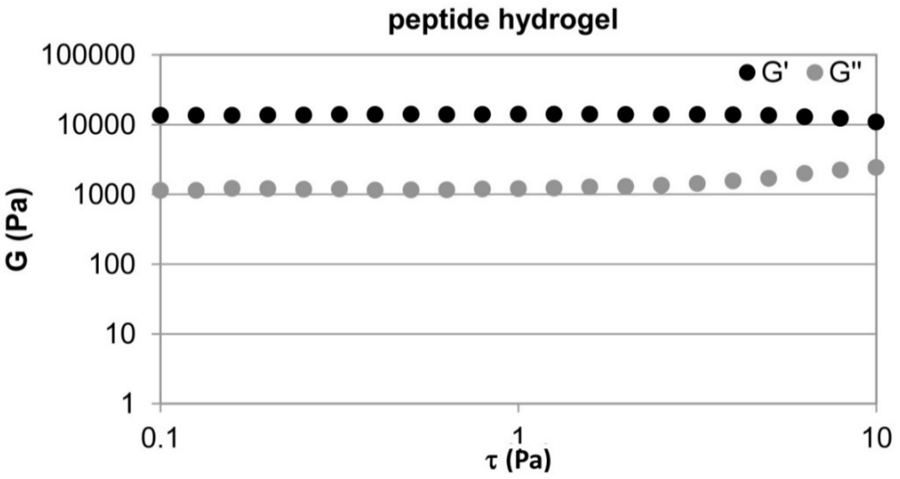

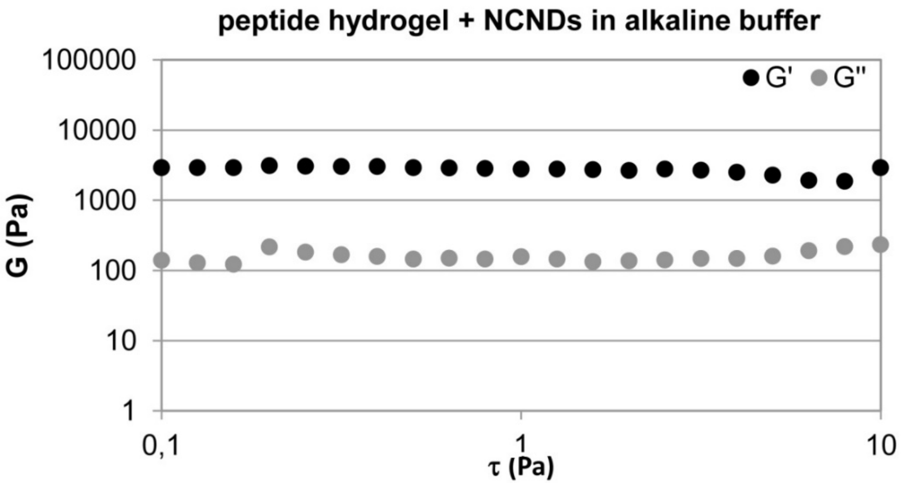

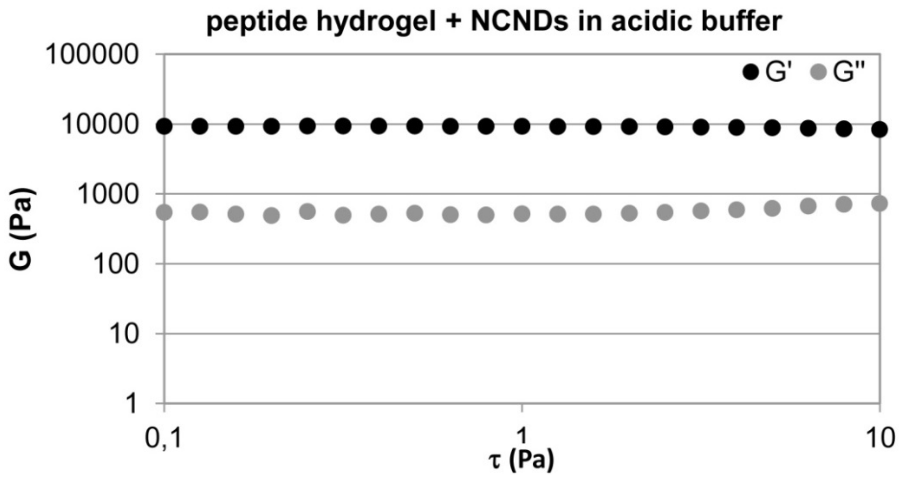

CD spectra

Note: Molar ellipticity in the graphs below (y axis) was divided by 1000.

Peptide in solution (alkaline buffer):

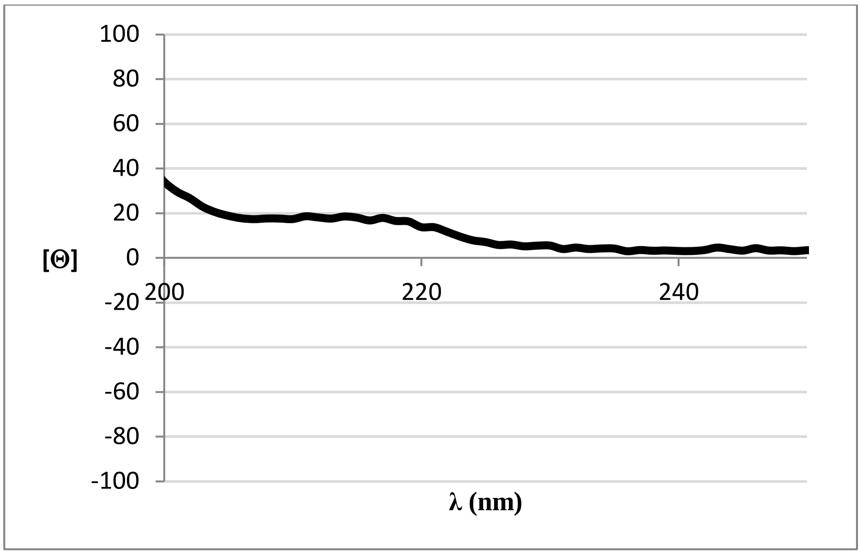

Peptide in solution + NCNDs (alkaline buffer):

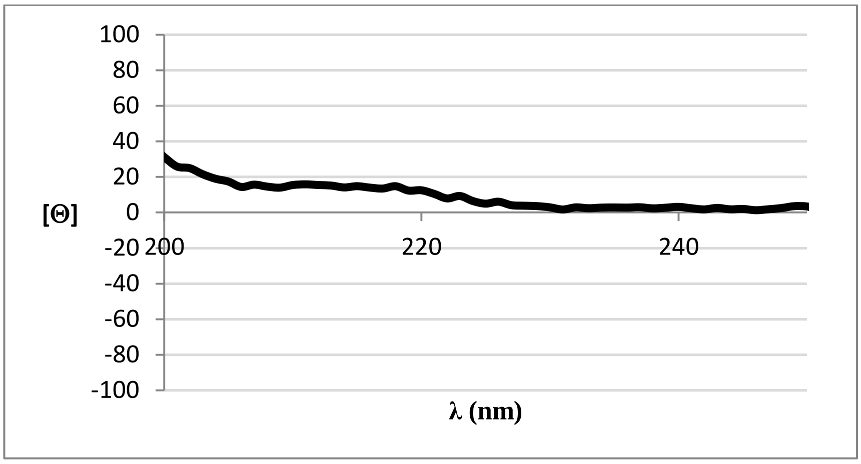

FT-IR

The FT-IR spectra were collected on a Perkin Elmer System 2000 with KBr discs in transmission mode. The scanned wavenumber range was from 4000 to 400 cm^{-1} with 16 accumulations at a resolution of 4 cm^{-1}. Freshly prepared samples were left to settle and gel for 24 hours in a glass vial. A small portion of the gel was then transferred on a clean glass slide, then dried under vacuum overnight. Dried samples were mixed with KBr powder and then pressed to obtain discs. Below are reported the FT-IR spectra.

Peptide hydrogel:

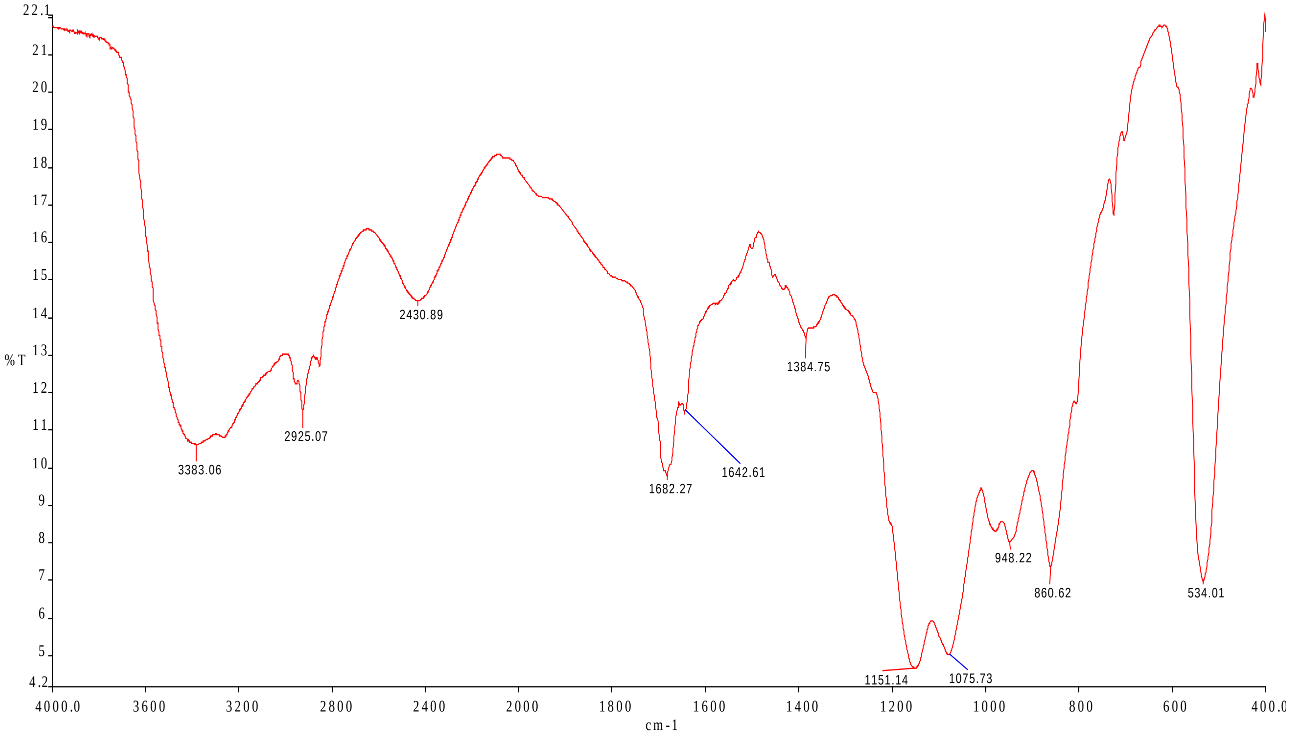

Peptide hydrogel with NCNDs in alkaline buffer:

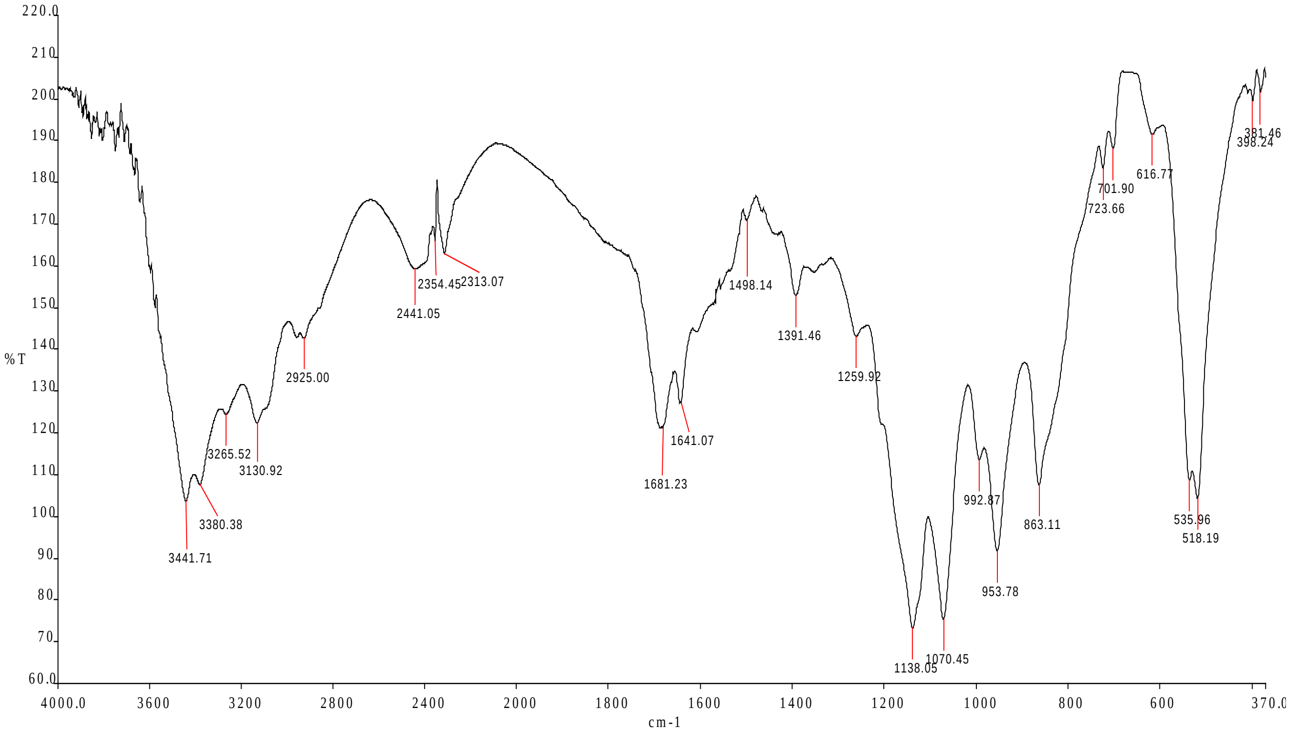

Peptide hydrogel with NCNDs in acidic buffer:

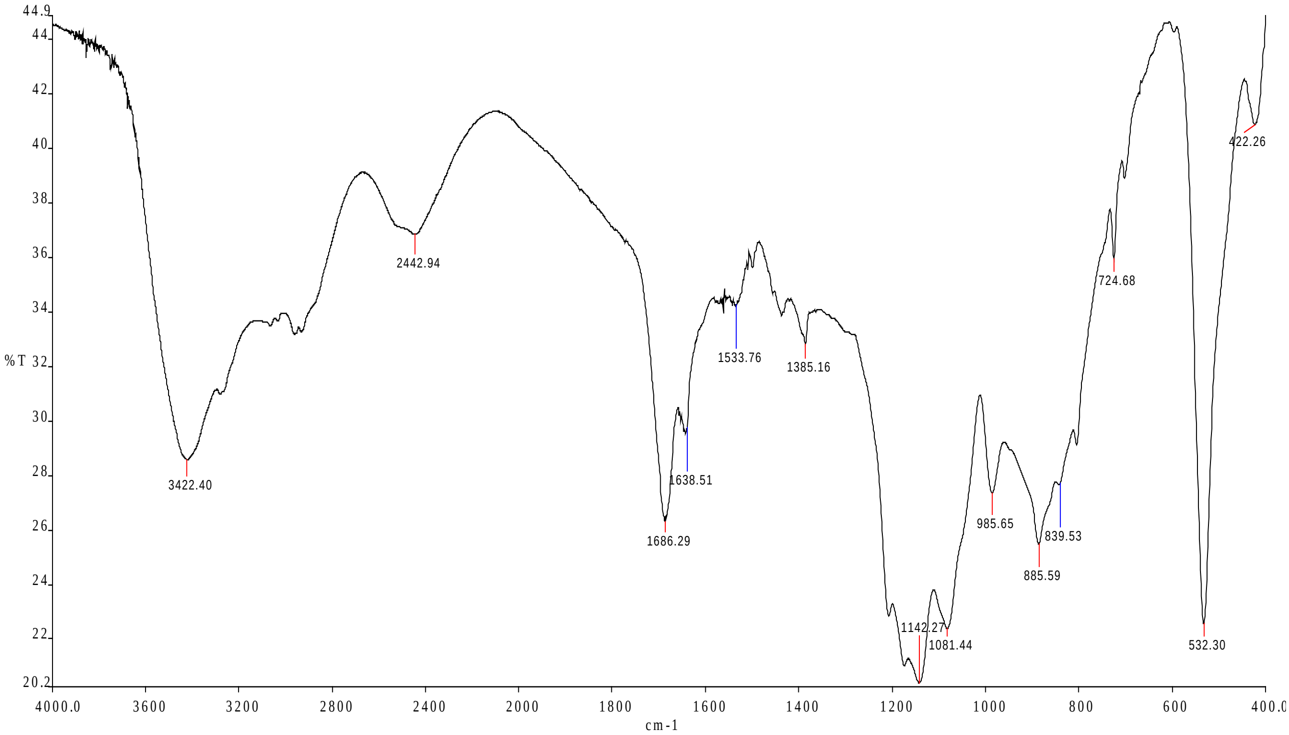

TEM images for gradual pH change experiment

Peptide alone:

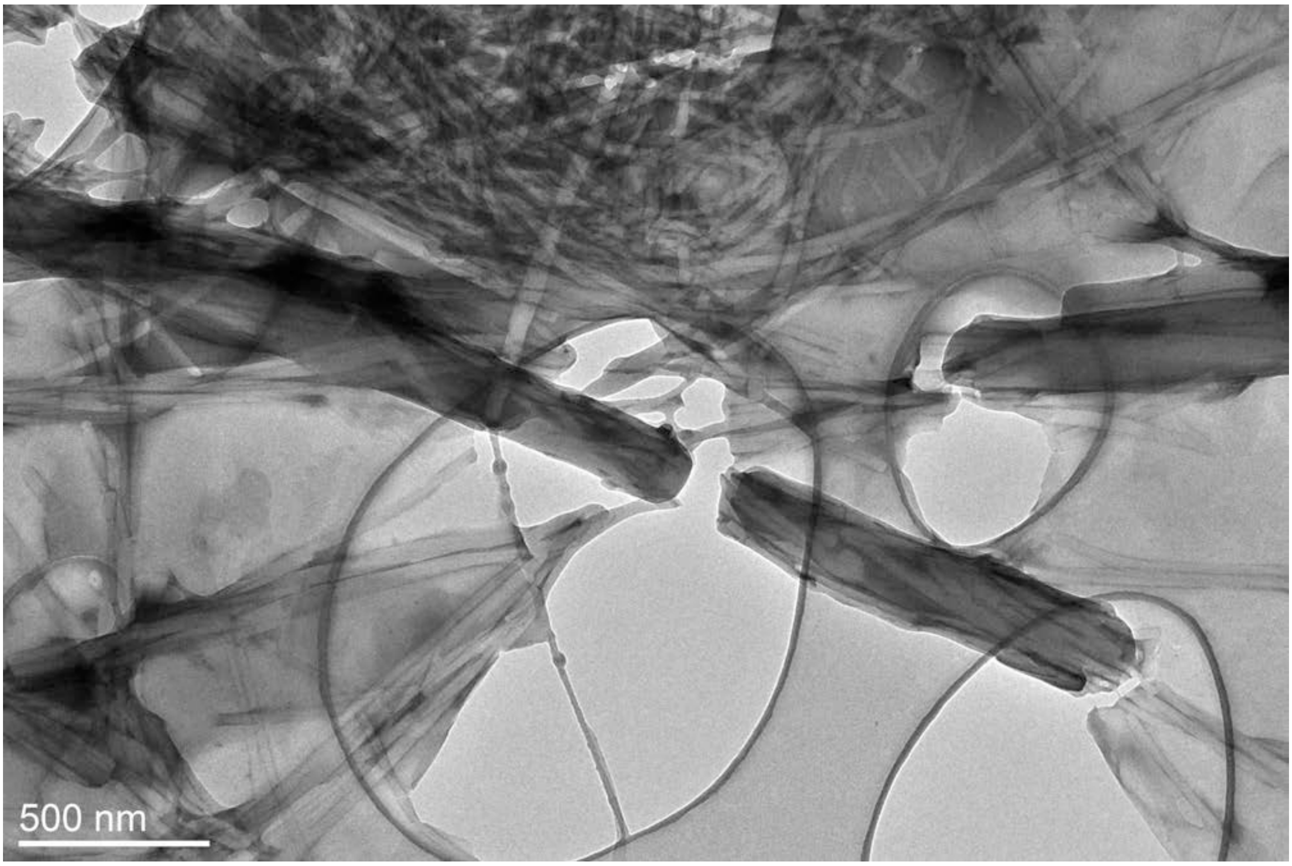

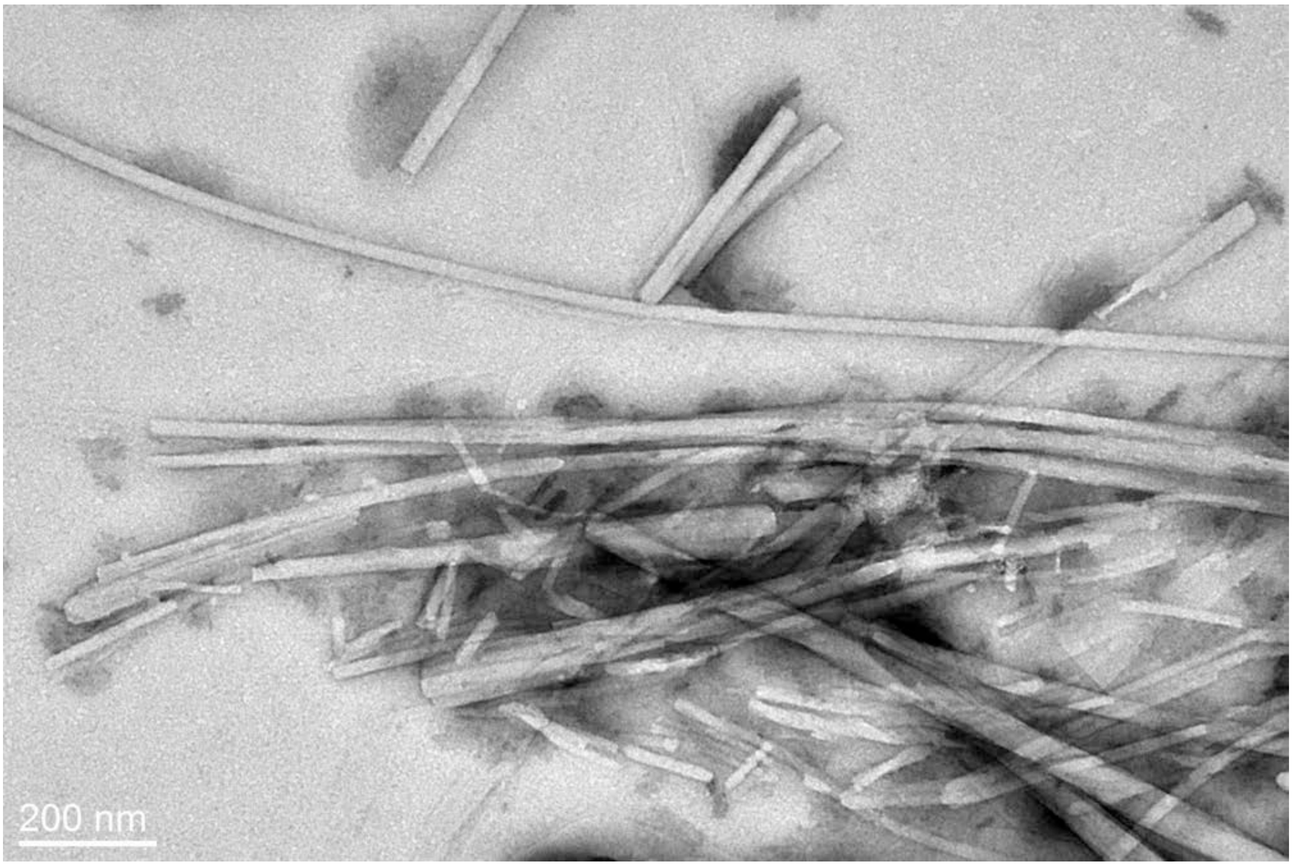

Peptide + NCND in alkaline buffer:

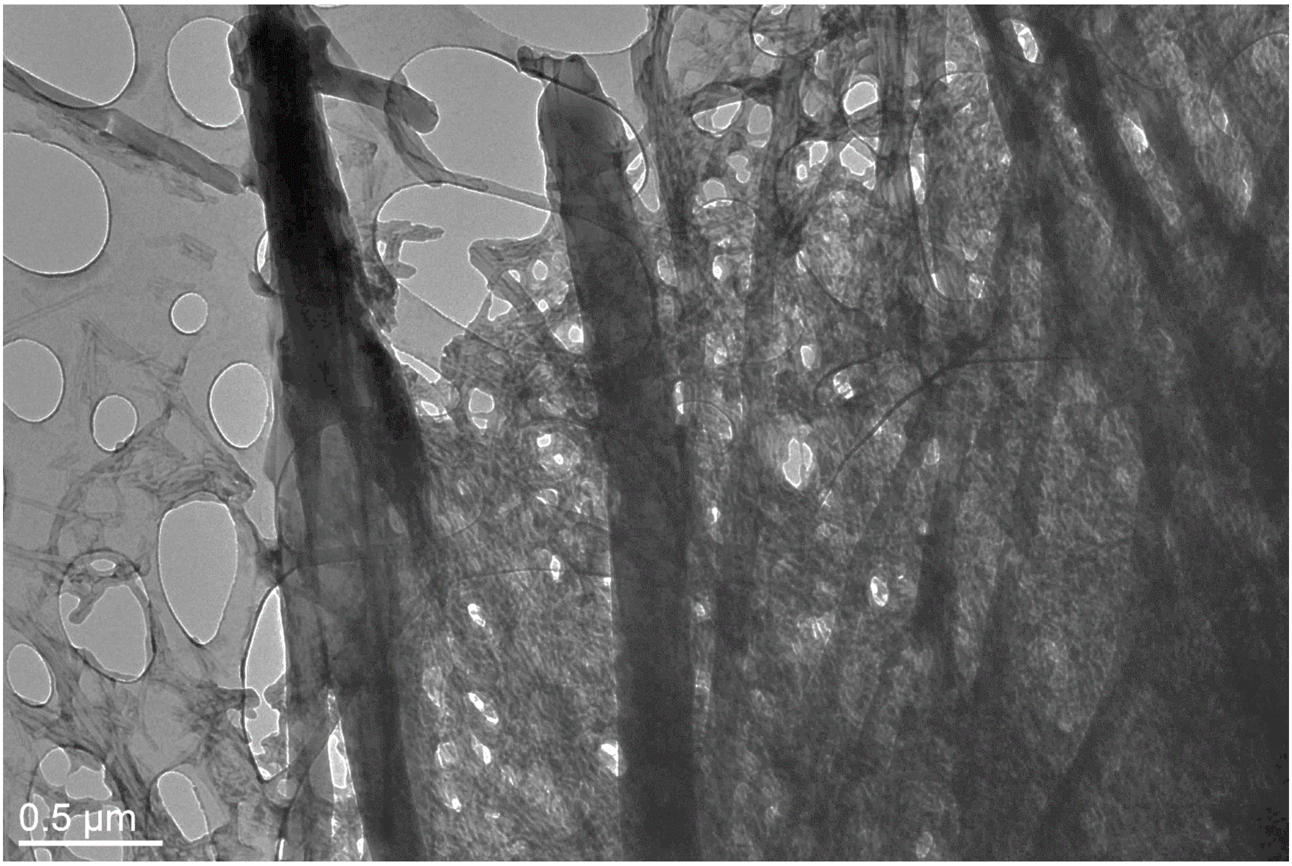

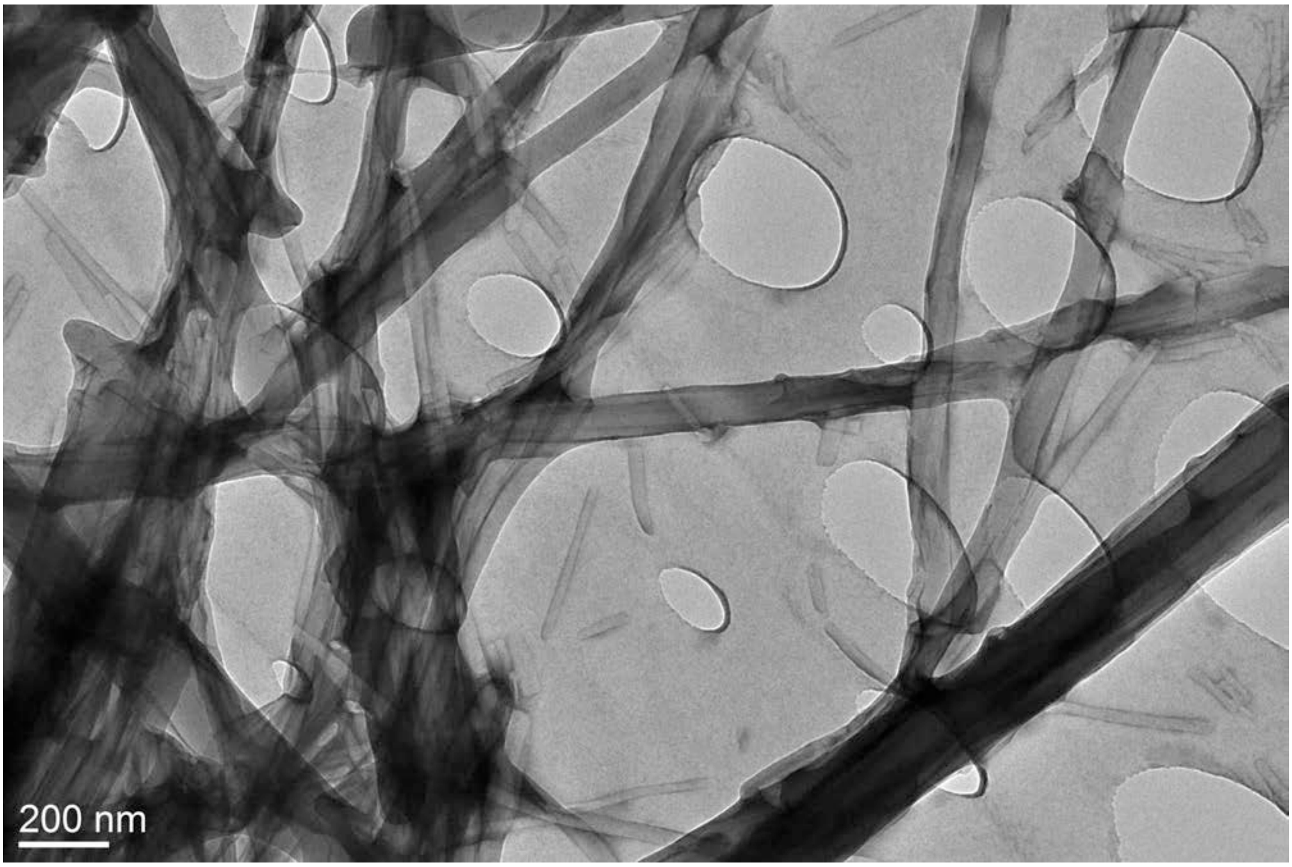

Peptide + NCND in acidic buffer:

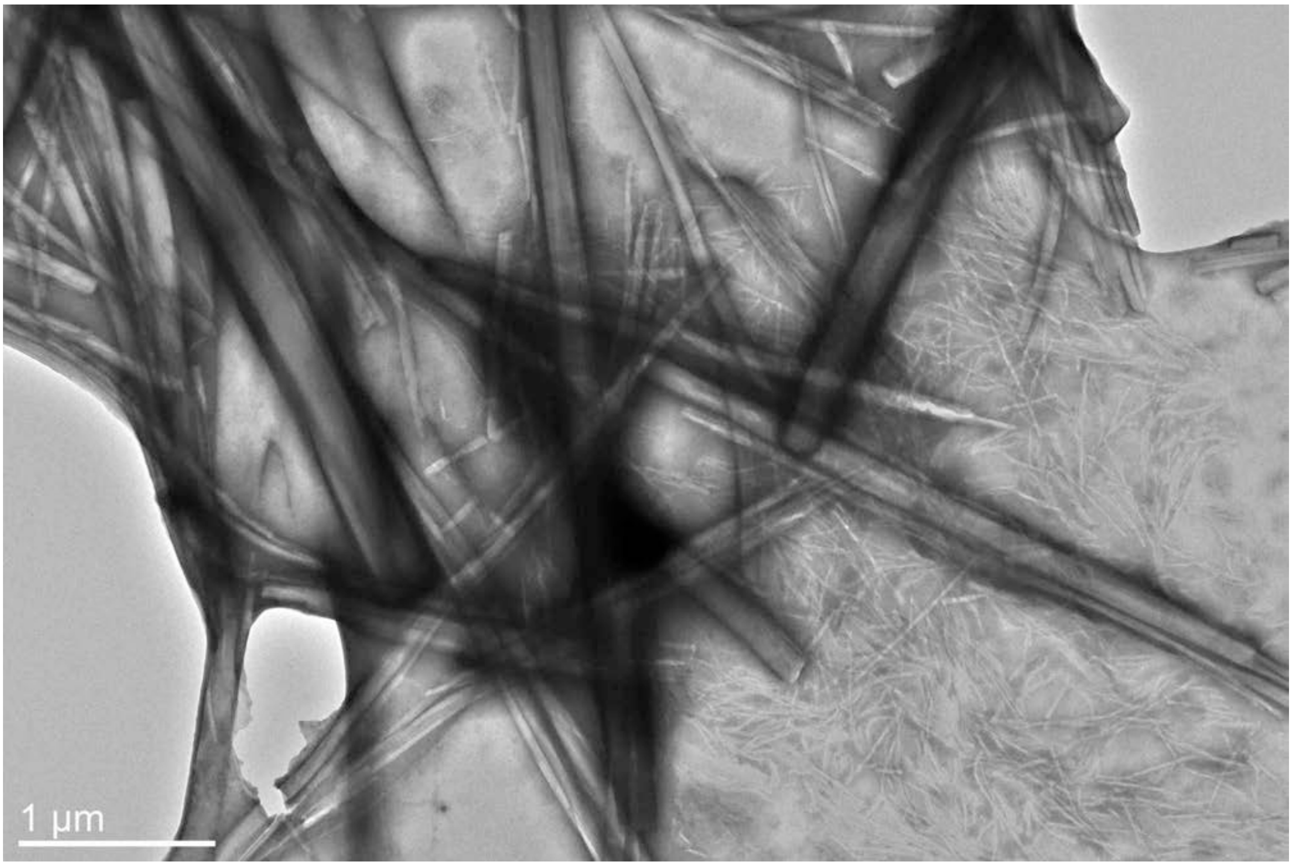

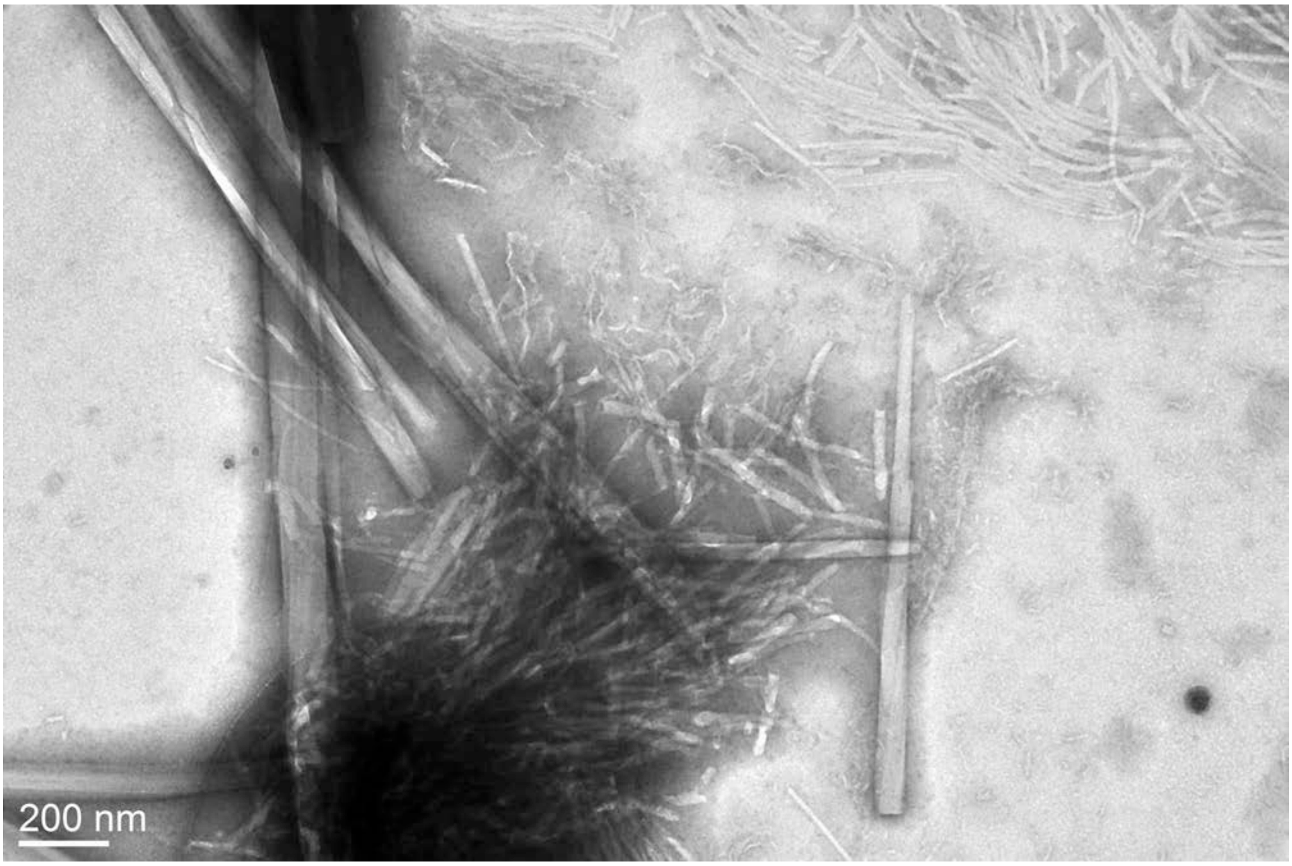

Circular dichroism with heating ramp

Note: Molar ellipticity in the graphs below (y axis) was divided by 1000.

Peptide alone:

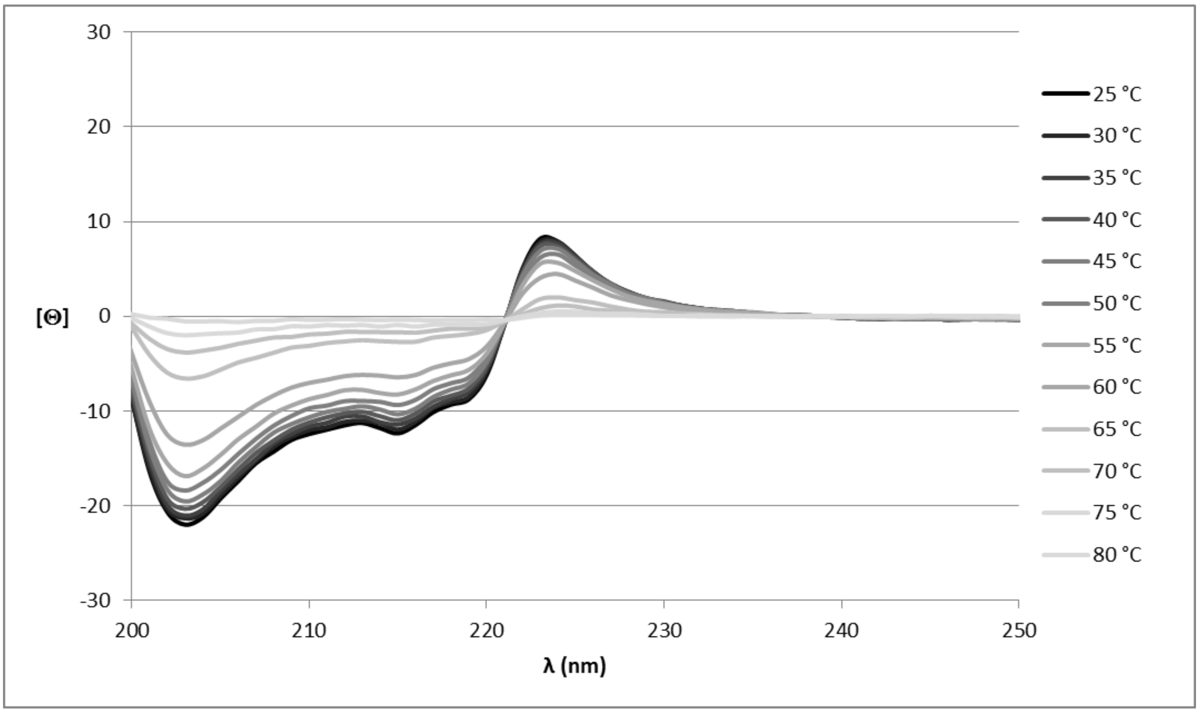

Peptide + NCND in alkaline buffer:

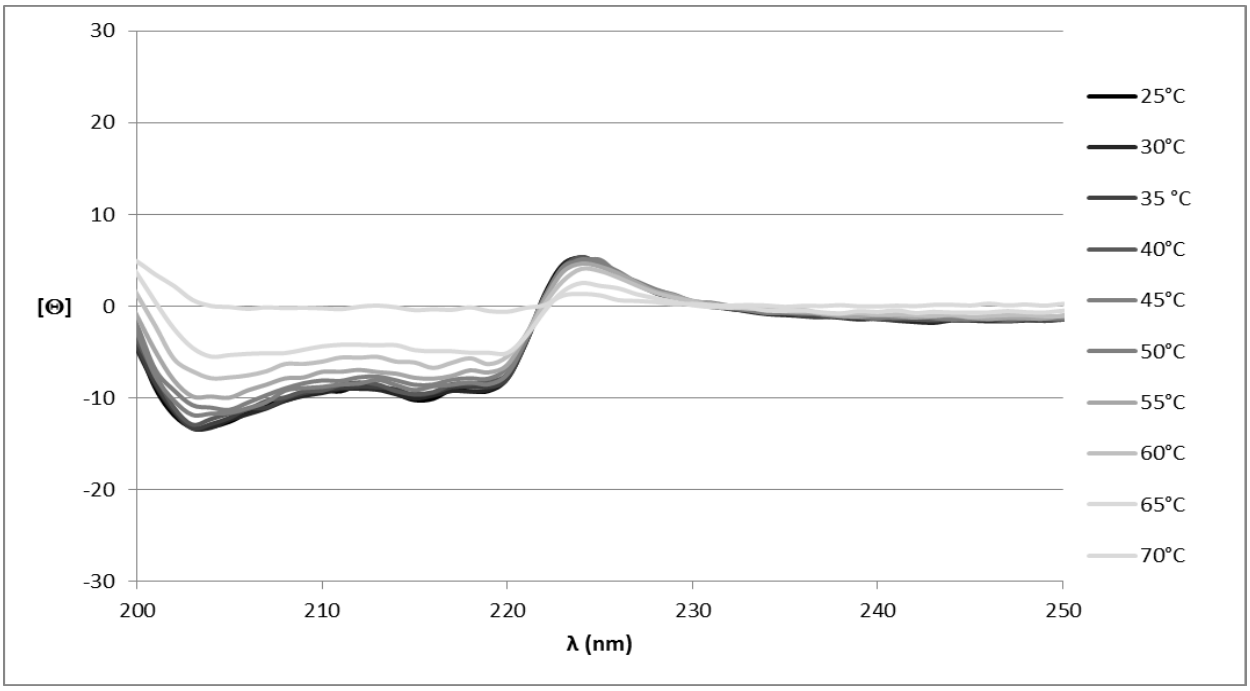

Peptide + NCND in acidic buffer:

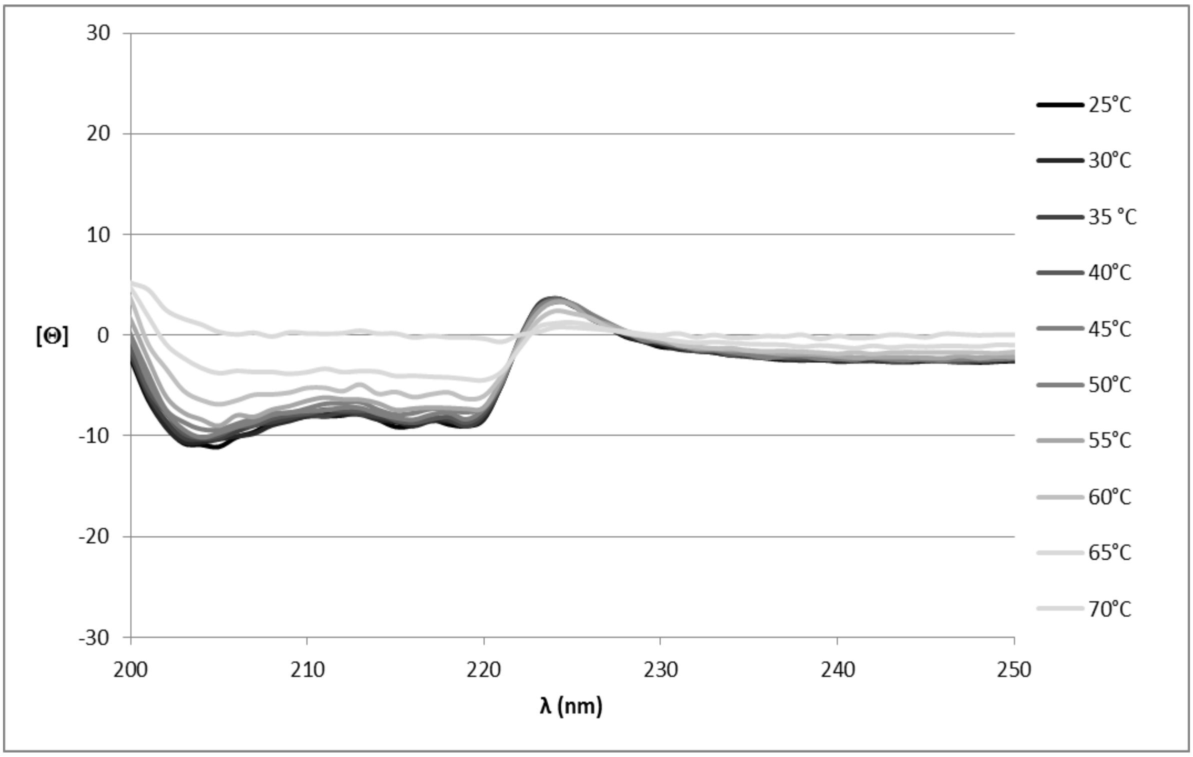

DSC

DSC data were collected on a Q100 calorimeter (TA Instruments). The hydrogel samples were prepared directly in the DSC pans. Pans were closed with their lids, and measurements started after 15 min at room temperature. DSC scans started with an isotherm at 20 °C for 10 min, followed by a 5 °C min^{-1} ramp up to 200 °C (i.e., below peptide decomposition temperature). Measurements were repeated at least in triplicates.

Peptide alone:

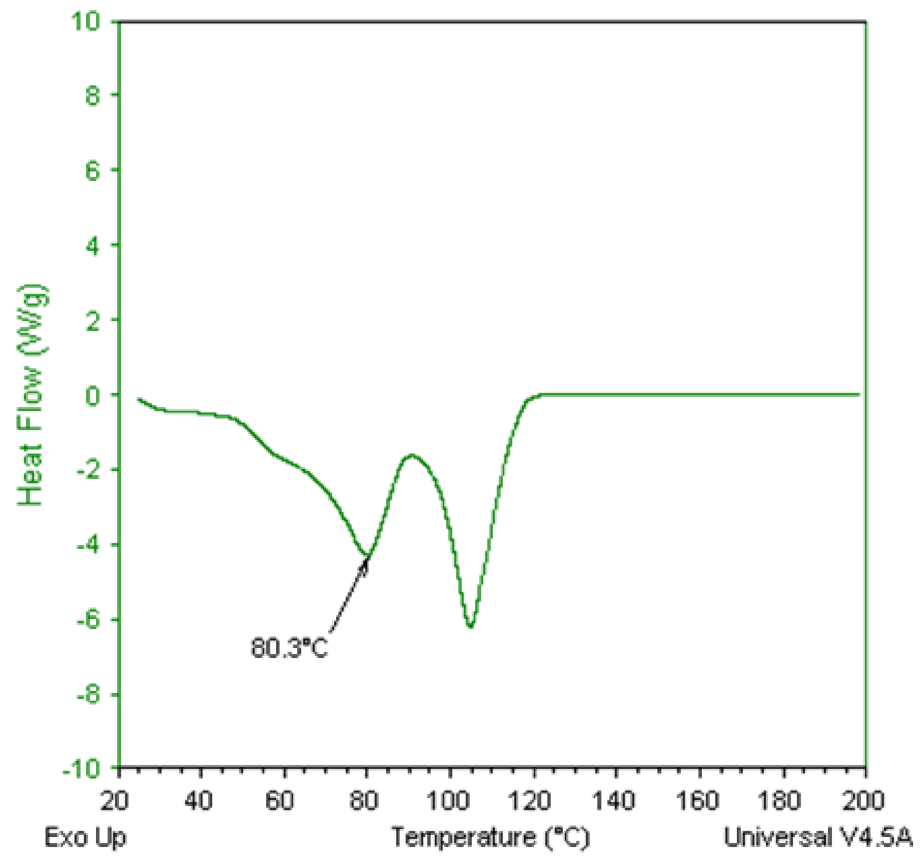

Peptide + NCND in alkaline buffer:

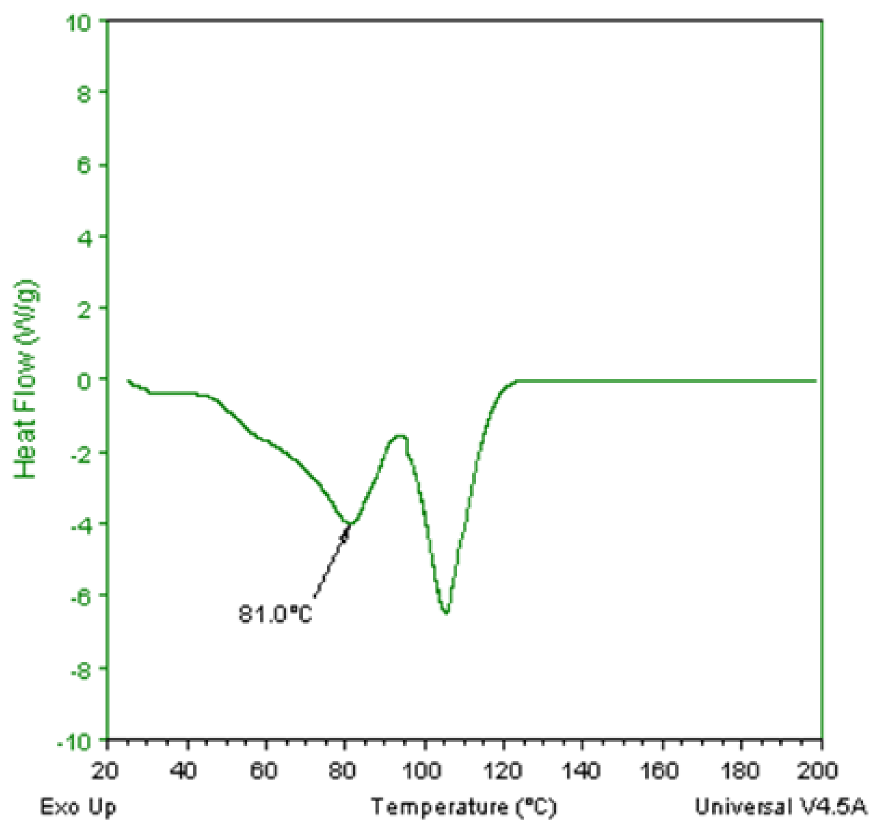

Peptide + NCND in acidic buffer:

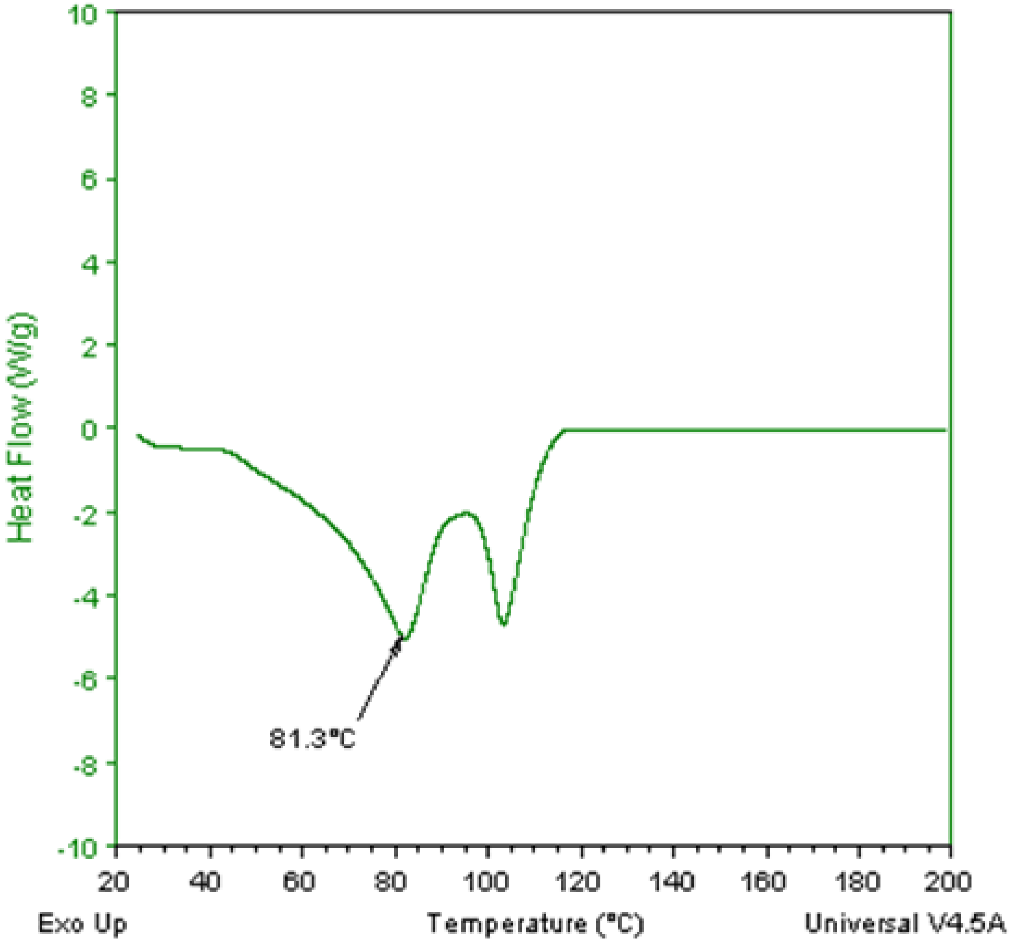

Supplement: File 1 — Additional experimental information. [file Beilstein_J_Nanotechnol-08-1553-s001.pdf]
